# Origin and Evolution of the Neo-Sex Chromosomes in Pamphagidae Grasshoppers through Chromosome Fusion and Following Heteromorphization

**DOI:** 10.3390/genes8110323

**Published:** 2017-11-13

**Authors:** Ilyas Yerkinovich Jetybayev, Alexander Gennadievich Bugrov, Olesya Georgievna Buleu, Anton Gennadievich Bogomolov, Nikolay Borisovich Rubtsov

**Affiliations:** 1 The Federal Research Center, Institute of Cytology and Genetics, Russian Academy of Sciences, Siberian Branch, Lavrentjev Ave., 10, 630090 Novosibirsk, Russia; mantisanton@gmail.com (An.G.B.); rubt@bionet.nsc.ru (N.B.R.); 2 Institute of Systematics and Ecology of Animals, Russian Academy of Sciences, Siberian Branch, Frunze str. 11, 630091 Novosibirsk, Russia; bugrov04@yahoo.co.uk (Al.G.B.); buleu.olesya@mail.ru (O.G.B.); 3 Novosibirsk State University, Department of Natural Sciences, Pirogov str., 2, 630090 Novosibirsk, Russia

**Keywords:** Pamphagidae, grasshoppers, neo-sex, chromosome, evolution, neo-X, neo-Y, Fluorescent in situ hybridization, chromosome microdissection, DNA libraries, chromosome painting

## Abstract

In most phylogenetic lineages, the evolution of sex chromosomes is accompanied by their heteromorphization and degradation of one of them. The neo-sex chromosomes are useful model for studying early stages of these processes. Recently two lineages of the neo-sex chromosomes on different stages of heteromorphization was discovered in Pamphagidae family. The neo-sex chromosome heteromorphization was analyzed by generation of DNA probes derived from the neo-Xs and neo-Ys followed with chromosome painting in nineteen species of Pamphagidae family. The homologous regions of the neo-sex chromosomes were determined in closely related species with the painting procedure and image analysis with application of the Visualization of the Specific Signal in Silico software package. Results of these analyses and distribution of C-positive regions in the neo-sex chromosomes revealed details of the heteromorphization of the neo-sex chromosomes in species from both phylogenetic lineages of Pamphagidae grasshoppers. The hypothetical mechanism of the neo-Y degradation was suggested. It includes expansion of different repeats from the proximal neo-Y chromosome region by inversions, spreading them towards distal region. Amplification of these repeats leads to formation of C-positive regions and elimination of the C-negative regions located between them.

## 1. Introduction

Sexual reproduction is one of the most important evolutionary acquisitions. Despite the disadvantage in number of potential progeny (so called twofold cost of males) [1], sexual organisms are evolutionarily successful due to genetic recombination and additional mechanisms for the negative genetic ballast elimination [2]. In animals, sexual reproduction is realized mainly through gonochorism, which implies sexual dimorphism and sex determination. Genetic factors regulating this determination usually include master switch genes involved in sexual differentiation. As soon as sexual factors arise, the chromosome harboring them can refer to a sex chromosome [3,4]. There are two main sex chromosome systems: XX♀/XY♂ and ZZ♂/ZW♀. In the earliest stages of their evolution, sex chromosomes are homomorphic. Later the heteromorphization takes place. This process usually includes the accumulation of constitutive heterochromatin and loss of euchromatic regions. Finally, one of the sex chromosomes can disappear altogether providing XX♀/X0♂ or ZZ♂/Z0♀ [5]. The degradation of sex chromosomes is still not well understood, however the most generally accepted modern theories on sex chromosome evolution are based on Muller’s ideas. They suggest the suppression of recombination in sex chromosomes as a crucial point in their evolution [3,6,7,8].

The majority of taxa are characterized by the stable sex chromosomes showing advanced heteromorphization [9]. The early stages of the sex chromosome evolution can be found in some fishes [10], reptilians [11], amphibians [12] and plants [13]. In some groups, the fusion of sex chromosome with an autosome leads to formation of the neo-sex chromosomes. These neo-sex chromosomes show similar heteromorphization processes in their further evolution, indicating that comparative cytogenetics of the species with the neo-sex chromosomes is a useful approach for studying the sex chromosome evolution. Studies involving taxa containing species with neo-sex chromosomes in different stages of heteromorphization can be especially fruitful.

Among insects, the detailed studies of the neo-sex chromosome evolution were performed in *Drosophila pseudoobscura*, *Drosophila miranda* and *Drosophila albomicans* [14,15]. The neo sex chromosomes in these species derived from different chromosome fusions, which took place at different time ago. In the evolutionary history of *D. pseudoobscura* the Y chromosome fusion with one of the autosomes took place approximately 13 million years ago (MYA). At some point, its homolog fused with the X chromosome forming the neo-X. To date the neo-Y chromosome has degraded significantly. Its morphology became similar to the Y of *D. melanogaster* but in these two species the Ys do not share any common single-copy genes [16,17]. Another neo-Y chromosome in the genus *Drosophila* arose approximately 2 MYA in the lineage of *D. miranda* [6]. The ancestral neo-Y fused with another autosome and its homolog formed the neo-X in addition to the ancestral X. The most recent event of the neo-sex chromosome formation in *Drosophila* took place approximately 0.12 MYA. The result of the X and the Y chromosome fusion with homologous autosomes can be observed in *D. albomicans* [18,19]. Thus, in *Drosophila* many neo-sex chromosome systems derived from the fusion of the ancestral Y with one of the autosomes. We should note that *Drosophila* male meiosis dispenses with recombination, synapsis and the associated structures that may determine and facilitate very fast evolution of the neo-Y chromosomes in all evolutionary lineages of fruit fly.

Among the grasshoppers, the neo-sex chromosomes were described in many groups. However, distribution of the species with neo-sex chromosomes between taxa is non-uniform. Two groups show high number of such species. The first group is grasshoppers of South America belonging to the Melanoplinae subfamily of Acrididae. Independent fusions of the X with autosomes took place in different phylogenetic lineages and various chromosome rearrangements including pericentric inversions in the neo-Y chromosomes were described in this group [20,21,22]. In Melanoplinae, there are species with evolutionary young and old neo-sex chromosomes. Their age was estimated according to the number and distribution of chiasmata in the sex bivalent. The old neo-sex chromosomes forms bivalents with lower number of chiasmata that are located within the smaller, often terminal part of it [22].

The second group of grasshoppers with neo-sex chromosomes is Pamphagidae family. In this family two lineages of grasshoppers with neo-sex chromosomes were discovered. In one lineage (Thrinchinae subfamily), the fusion of the X chromosome with an autosome probably took place recently. The neo-Y is similar to original autosome according to the size. In the second lineage (Nocarodeini tribe of Pamphaginae subfamily), the neo-sex chromosomes exhibit advanced stages of heteromorphization with significant degradation of the neo-Y [23,24]. This study is devoted to the comparative analysis of the neo-sex chromosomes in both evolutionary lineages in Pamphagidae by the generation of microdissected DNA probes from neo-sex chromosomes or their regions, followed by in situ hybridization with chromosomes of different species of Pamphagidae. The possible mechanisms of the neo-sex chromosome heteromorphization are discussed.

## 2. Materials and Methods

### 2.1. Samples of Pamphagidae Grasshoppers

**Figure 1:**
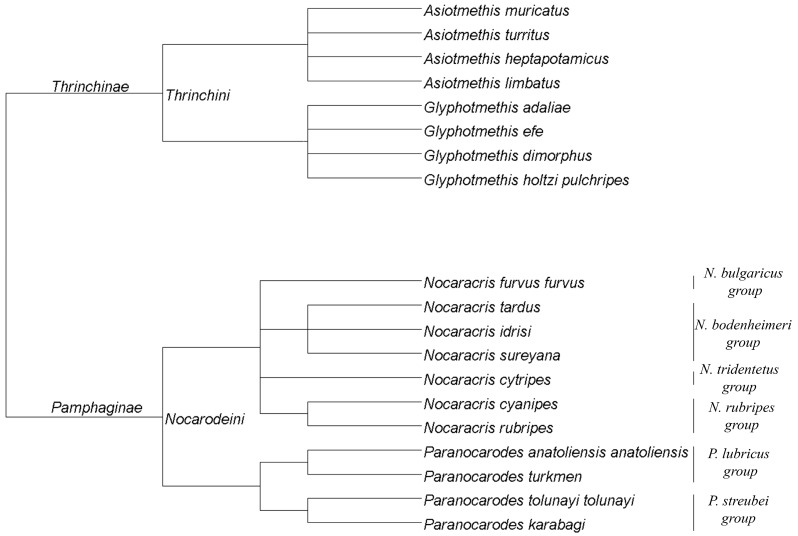
Cladogram of studied species according to the nomenclature proposed by Ünal [25].

Samples of 8 species from Thrinchinae subfamily and 11 species from Pamphaginae subfamily were collected during summer seasons of 1994, 1995, 2013, 2014 and 2016 in Bulgaria, Armenia, Turkey and Kazakhstan (Table 1). In the paper, the new nomenclature of Pamphagidae grasshoppers proposed by Ünal [25] was applied. The cladogram based on the data published by Ünal [25] is shown on Figure 1. We should note that in the previous paper [24] we used old nomenclature for Pamphagidae grasshoppers.

### 2.2. Chromosome Preparation

In all studied species, the males were used for meiotic chromosome preparation according to standard procedures. In *Nocaracris furvus*, females were kept in the cage with moist sand for oviposition. Eggs were used for mitotic chromosome preparation. Chromosome preparation for fluorescence in situ hybridization and chromosome microdissection was performed as described earlier [26].

### 2.3. Microdissected DNA Library and DNA Probe Preparation

Microdissection of sex chromosomes was performed according to described protocol [27] with some modifications. For each DNA probe, 15–20 copies of the chromosome or chromosome region were collected by extended glass needle using a micromanipulator MR (Zeiss, Oberkochen, Germany). Proteinase K treatment and low temperature cycles of degenerate oligonucleotide-primed polymerase chain reaction (DOP-PCR) with Sequenase 2.0 (ThermoFisher Scientific, Waltham, MA, USA) were performed exactly as described [27]. High temperature cycles of DNA amplification were carried out with Encyclo Polymerase mix (Evrogen, Moscow, Russia) in 33 cycles of PCR according to the producer’s recommendation. DNA probes were labeled in an additional 25 cycles of PCR with Tamra-5-dUTP and Fluorescein-12-dUTP (Biosan, Novosibirsk, Russia) as described [28].

### 2.4. Fluorescence In Situ Hybridization

Two-color Flourescent *in situ* hybridization (FISH) was carried out as described [29] with small modifications. Chromosome preparations were denaturated in 70% formamide/2× SSC solution for 5 min at 75 °C and dehydrated in standard series of precooled ethanol at −20 °C. The DNA probes were dissolved in hybridization mixture and denaturated separately for 5 min at 95 °C. Then DNA probes were incubated for 60 min at 37 °C for repetitive DNA renaturation. Hybridization of DNA probes on meiotic chromosomes was carried out overnight in the moist chamber at 37 °C. After hybridization, slides were washed at low stringency condition (3 times for 5 min in 50% Formamide/2× SSC at 45 °C; 3 times for 5 min in 2× SSC at 45 °C and 3 times for 5 min in 0.2× SSC at 45 °C). DAPI counterstaining was preformed after FISH using Vectashield Antifade Mounting Medium containing 4′,6-diamidino-2-phenylindole (DAPI) (Vector labs, Burlingame, CA, USA) applied directly under a coverslip which was then sealed with rubber cement.

### 2.5. Microscopy

Microscopic analysis was carried out at the Centre for Microscopy of Biological Objects (Institute of Cytology and Genetics, Novosibirsk, Russia). Chromosomes were analyzed with an AxioImager.M1 (Zeiss) fluorescence microscope equipped with #49, #46HE, #43HE filter sets (Zeiss), ProgRes MF CCD camera (JenaOptik, Jena, Germany). Software package ISIS5 (MetaSystems GmbH, Altlussheim, Germany) was used for image capture and analysis.

### 2.6. Image Analysis by the Visualization of the Specific Signal In Silico Software

Two-color FISH microscopic images were additionally analyzed with the Visualization of the Specific Signal In Silico (VISSIS) software package [30] for detection of the FISH signal produced with chromosome specific DNA sequences. The VISSIS software uses algorithms of determination of FISH signal derived from chromosome specific DNA sequences by elimination of the FISH signal from dispersed repeats. Two microdissected DNA probes from different chromosomes are necessary for this analysis. Both microdissected DNA probes used for two-color FISH contain chromosome specific DNA sequences and interspersed repeats present in all chromosomes of studied species. The VISSIS software performs normalization of the FISH signals derived from both DNA probes and then subtracts the signal of the second DNA probe from the signal of the chromosome specific DNA probe. The final image after the VISSIS analysis is similar to result of chromosome in situ suppression hybridization (CISS-hybridization) [30] but in contrast to CISS-hybridization the VISSIS analysis does not eliminate signal derived from chromosome specific repeats liked repeats enriched the C-positive chromosome regions.

### 2.7. Chromosome Nomenclature

The nomenclature of Pamphagidae grasshopper chromosomes [31] was used for the description of chromosomes and karyotypes in studied species. The neo-sex chromosome nomenclature was proposed by White [5]. According to it, the XL arm of the neo-X refers to the ancestral X, while the XR refers to the arm derived from an autosome.

## 3. Results

### 3.1. Microdissected DNA Probes Derived from Neo-Sex Chromosomes

Microdissected DNA probes were generated from the neo-sex chromosomes of the five species, belonged to two independent evolutionary lineages in Pamphagidae family. For the first evolutionary lineage (Thrinchini tribe, Thrinchinae subfamily), the set of DNA probes was obtained from short arm of the neo-X chromosome (the XL) that represents the ancestral X chromosome (*AheXl* DNA probe) and the proximal part of the neo-Y chromosome (*AheYcen* DNA probe) of *Asiotmethis heptapotamicus*. Sex chromosome bivalent was easily recognizable in the prophase and first metaphase of meiosis. However, due to conjugation between the neo-Y and the XR almost in all chromosome spreads only proximal part of the neo-Y chromosome could be dissected from the sex bivalent without contamination with the regions of the XR. For the second evolutionary lineage (Nocarodeini tribe, Pamphaginae subfamily), the species for microdissected DNA probe generation were selected to include representatives of all studied genera. DNA probes were generated from short arm of the neo-X chromosome (the XL) and the whole neo-Y chromosome of four species, namely *Nocaracris cyanipes* (DNA probes: *NcyXl*, *NcyY*), *Nocaracris tardus* (DNA probes: *NtaXl*, *NtaY*), *Nocaracris rubripes* (DNA probes: *NruXl*, *NruY*) and *Paranocarodes tolunayi tolunayi* (DNA probes: *PtoXl*, *PtoY*). In this lineage, the neo-Y chromosome frequently forms only terminal chiasma and can easily be dissected from the sex bivalent. Two-color FISH performed with these pairs of DNA probes allowed applying the VISSIS software for reduction of FISH signal produced with interspersed repeats.

**Figure 2:**
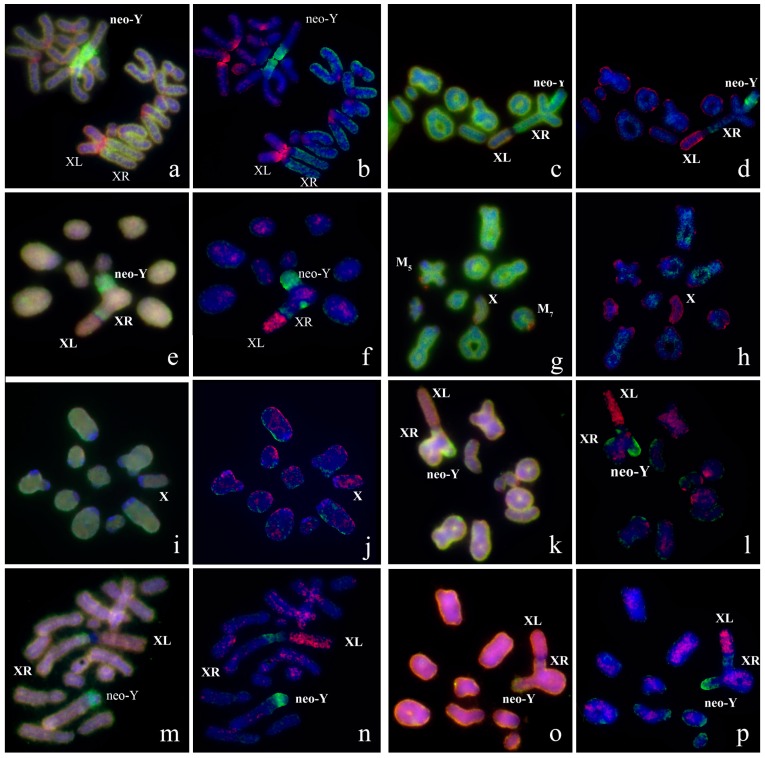
FISH (**a**,**c**,**e**,**g**,**i**,**k**,**m**,**o**) of *AheXL* (red) and *AheYcen* (green) DNA probes with meiotic chromosomes of Trinchinae species and VISSIS analysis (**b**,**d**,**f**,**h**,**j**,**l**,**n**,**p**) of microphotographs. XL indicates short arm of neo-X that corresponds to ancestral X chromosome, XR indicates long arm of neo-X chromosome derived from autosome fusion. Neo-Y, M_5_ M_7_ indicates corresponding chromosomes: (**a**,**b**) *Asiotmethis heptapotamicus songoricus*; (**c**,**d**) *Asiotmethis turritus*; (**e**,**f**) *Asiotmethis limbatus*; (**g**,**h**) *Asiotmethis muricatus*; (**i**,**j**) *Glyphotmethis adaliae*; (**k**,**l**) *Glyphotmethis holtzi pulchripes*; (**m**,**n**) *Glyphotmethis efe*; (**o**,**p**) *Glyphotmethis dimorphus dimorphus*.

**Figure 3:**
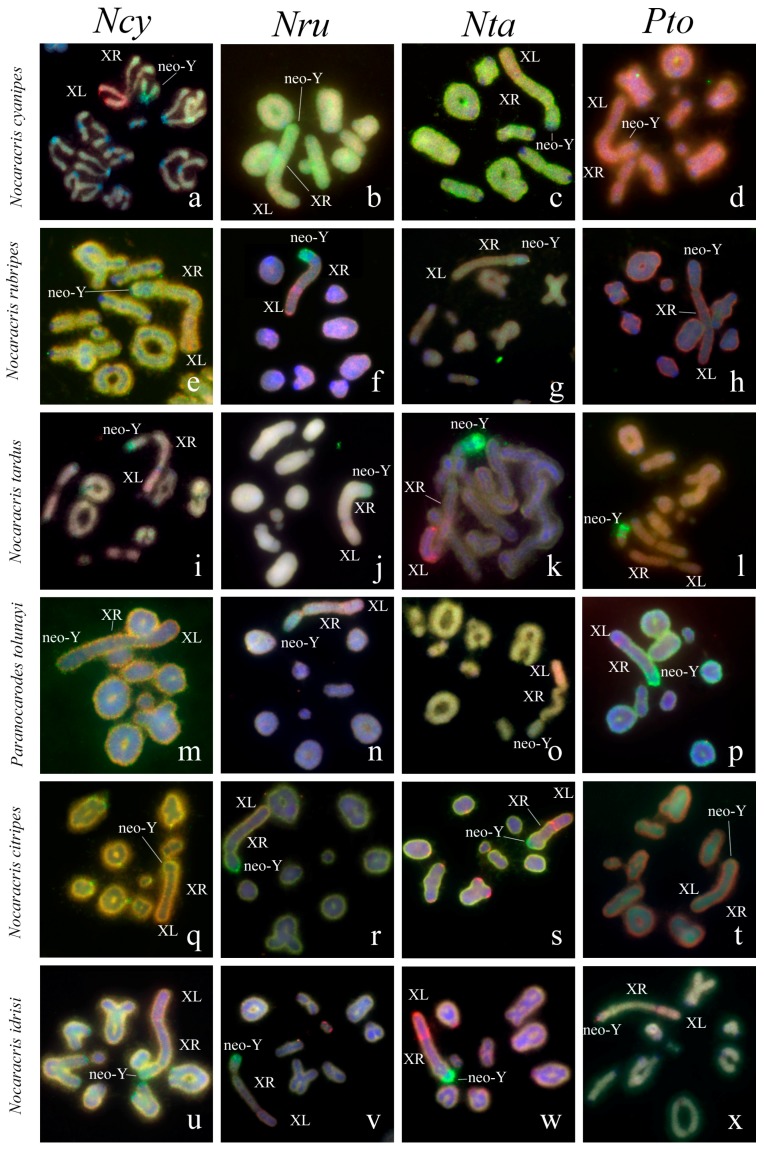
FISH of microdissected DNA probes with meiotic chromosomes of Nocarodeini species: the XL DNA probes (red) and the neo-Y DNA probes (green). XL indicates short arm of neo-X that corresponds to ancestral X chromosome, XR indicates long arm of neo-X chromosome derived from autosome fusion. Neo-Y indicates corresponding chromosome. Names of species indicated in rows and name of probes indicated in columns. (**a**–**d**) *Nocaracris cyanipes*; (**e**–**h**) *Nocaracris rubripes*; (**i**–**l**) *Nocaracris tardus;* (**m**–**p**) *Paranocarodes tolunayi tolunayi*; (**q**–**t**) *Nocaracris citripes citripes;* (**u**–**x**) *Nocaracris Idrisi*; (**a**,**c**,**e**,**g**,**i**,**k**,**q**,**s**,**u**,**w**) Intensive FISH signals from neo sex chromosome probes can be observed in C-positive regions of autosomes.

The obtained DNA probes were characterized by reverse painting. For each species, not less than 10 cells were analyzed. Intensity of FISH signals varied between chromosomes and chromosome regions. We discriminated four types of intensity that corresponded to different DNA content of the regions (Figure 2a and Figure 3a,f,k,p):

1. FISH signal of background intensity was usually observed in C-positive regions of some autosomes containing no interspersed repeats. It indicated to the absence of homology between DNA of chromosome region and DNA probe (Figures S5 and S6);
2. FISH signal produced by interspersed repeats was usually observed in C-negative regions of the autosomes. The intensity of the signal was slightly but distinctive higher than background level. It was decreased by the VISSIS software analysis (Figures S5 and S6);
3. Specific FISH signal that was stronger than signal produced by interspersed repeats. The signal was observed in C-negative regions of dissected chromosome or chromosome region after reverse painting. It was produced by hybridization of both unique DNA sequences from region of dissection and interspersed repeats (Figures S5 and S6);
4. Strong specific FISH signal was observed in C-positive regions of dissected chromosomes after reverse painting. We should note that strong FISH signal was observed also in C-positive regions of some autosomes. It was registered in region containing clustered repeats homologous to repeats of microdissected chromosome (Figures S5 and S6).

Application of the VISSIS software improved the differentiation of signal types (Figure 2b). The determination of C-negative region of dissection after reversed painting was similar to results of CISS-hybridization in mammals but intensity of chromosome specific FISH signal in C-positive regions in original chromosome was not suppressed.

### 3.2. Chromosome Painting in Pamphagidae Grasshoppers

**Figure 4:**
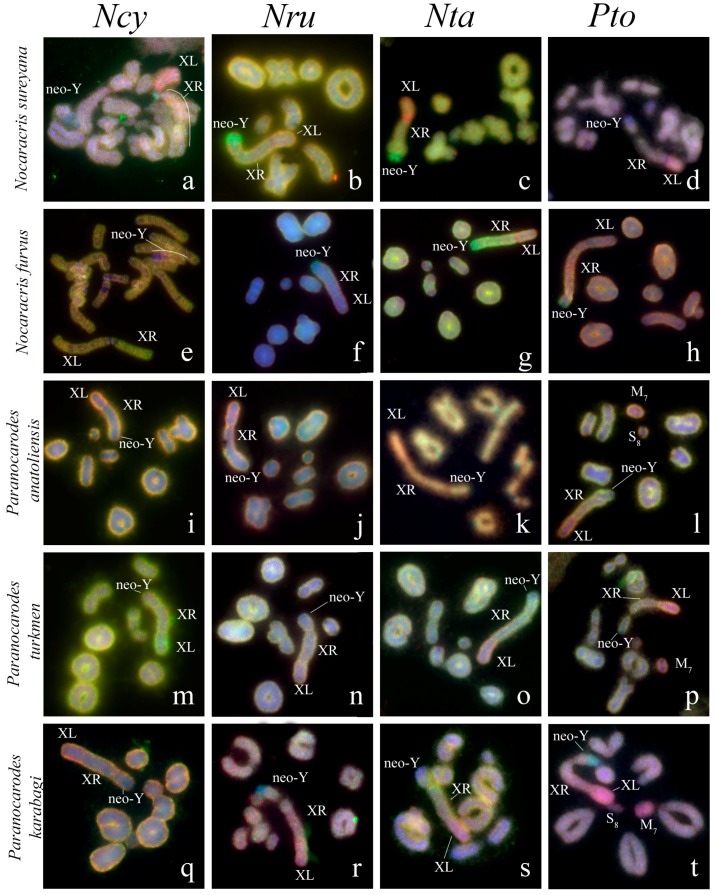
FISH of microdissected DNA probes with meiotic chromosomes of Nocarodeini species: the XL DNA probes (red) and the neo-Y DNA probes (green). XL indicates short arm of neo-X that corresponds to ancestral X chromosome, XR indicates long arm of neo-X chromosome derived from autosome fusion. Neo-Y indicates corresponding chromosome Names of species indicated in rows and name of probes indicated in columns. (**a**–**d**) *Nocaracris sureyana*; (**e**–**h**) *Nocaracris furvus*; (**i**–**l**) *Paranocarodes anatoliensis anatoliensis;* (**m**–**p**) *Paranocarodes turkmen*; (**q**–**t**) *Paranocarodes karabagi*; (**a,c,e,g**) Intensive FISH signals from neo sex chromosome probes can be observed in C-positive regions of autosomes.

**Figure 5:**
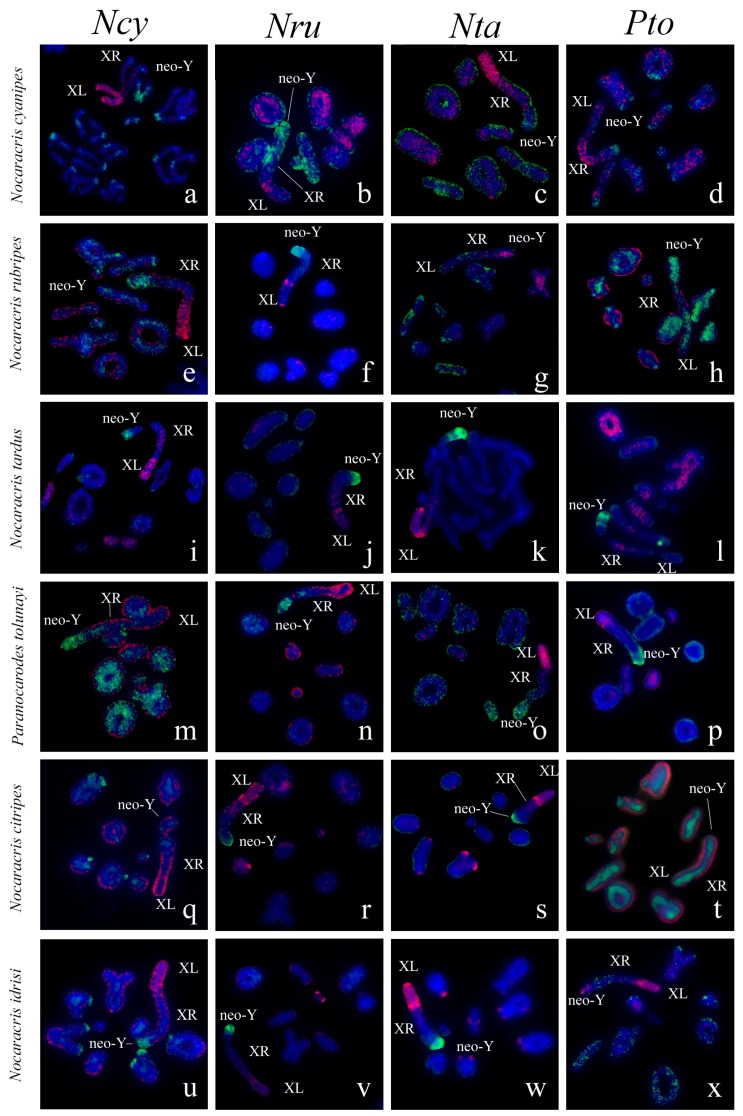
Results of the VISSIS analysis of the chromosome plates shown on the Figure 3. XL indicates short arm of neo-X that corresponds to ancestral X chromosome, XR indicates long arm of neo-X chromosome derived from autosome fusion. Neo-Y indicates corresponding chromosome. Names of species indicated in rows and name of probes indicated in columns. (**a**–**d**) *Nocaracris cyanipes*; (**e**–**h**) *Nocaracris rubripes*; (**i**–**l**) *Nocaracris tardus;* (**m**–**p**) *Paranocarodes tolunayi tolunayi*; (**q**–**t**) *Nocaracris citripes citripes;* (**u**–**x**) *Nocaracris idrisi*.

**Figure 6:**
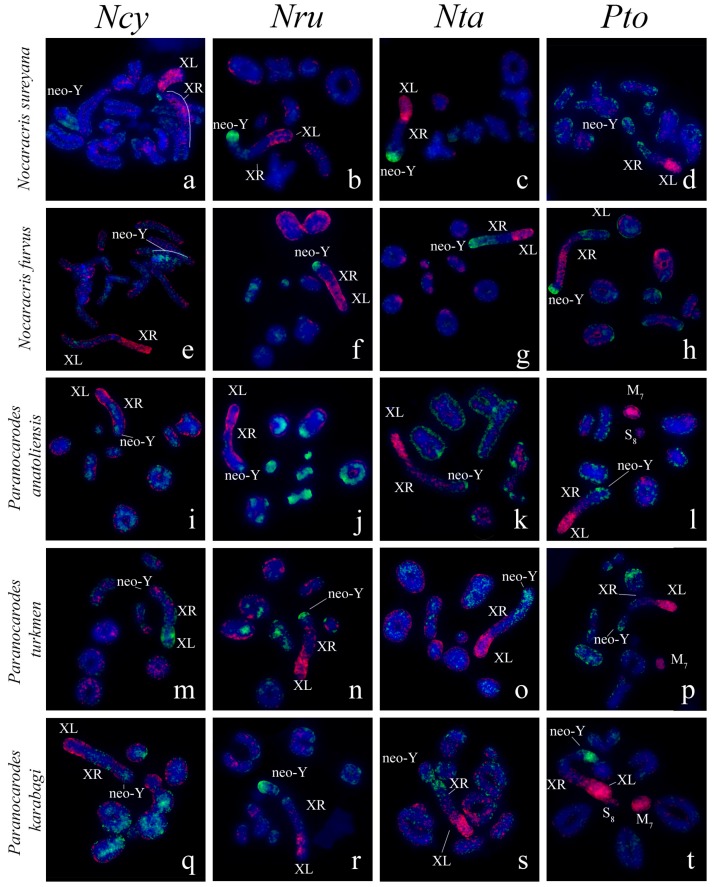
Results of VISSIS analysis of the chromosome plates shown on the of Figure 4. XL indicates short arm of neo-X that corresponds to ancestral X chromosome, XR indicates long arm of neo-X chromosome derived from autosome fusion. Neo-Y indicates corresponding chromosome Names of species indicated in rows and name of probes indicated in columns. (**a**–**d**) *Nocaracris sureyana*; (**e**–**h**) *Nocaracris furvus*; (**i**–**l**) *Paranocarodes anatoliensis anatoliensis;* (**m**–**p**) *Paranocarodes turkmen*; (**q**–**t**) *Paranocarodes karabagi*.

We performed FISH with microdissected DNA probes on chromosomes of 19 species from Pamphagidae family. The *AheXl* and *AheYcen* DNA probes were hybridized on chromosomes of 8 species from Thrinchinae subfamily (Figure 2) and 5 species from Nocarodeini tribe of Pamphaginae subfamily. FISH with four pairs of DNA probes generated from the neo-sex chromosomes of Nocarodeini species was performed on chromosomes of 11 species of Pamphaginae subfamily: 7 species belonged to Nocaracris genus and 4 species belonged to Paranocarodes genus (Figure 3 and Figure 4). The *NcyXl* and *NcyY* DNA probes were also hybridized with chromosomes of *Asiotmethis muricatus* from Thrinchinae subfamily. The analysis of microscopic images with the VISSIS software improved determination of specific FISH signal in chromosome regions in all cross-hybridization experiments (Figure 2, Figure 5 and Figure 6). Chromosomes painting of closely related species (species belonged to the same lineage) performed with microdissected DNA probes painted homologous C-negative and C-positive regions in chromosomes of these species. However, chromosome painting in Nocarodeini species with *AheXl* and *AheYcen* DNA probes and chromosome painting in Thrinchinae species with *NcyXl* and *NcyY* DNA probes failed to reveal homologous chromosome regions. Chromosome painting with *AheXl* and *AheYcen* DNA probes in species with XX/X0 sex chromosome system (*A. muricatus* and *Glyphotmethis adaliae*) was weaker than in the neo-XX/neo-XY Thrinchinae species (Figure 2g–j).

**Figure 7:**
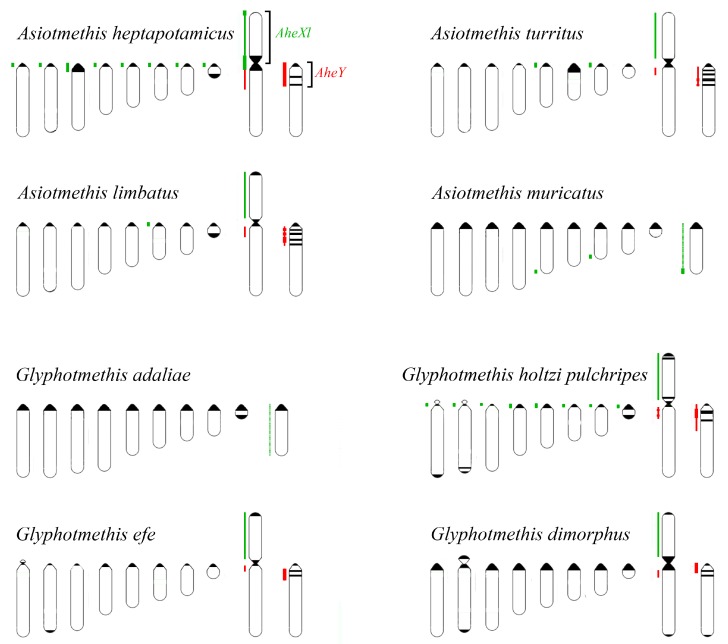
Schematic representation of chromosome painting results in Trinchinae species with *AheXl* (green) and *AheYcen* (red) DNA probes. The thick line shows intensive painting, while thin line shows less intensive painting. Black brackets indicate regions of microdissection. Distribution of C-positive regions on ideograms (black) is shown according to described earlier [23,24].

Painting with the *AheXl* DNA probe revealed homology of the XLs and Painting with the *AheYcen* probe revealed homologous region in proximal part of the long arm of the neo-X chromosome and in proximal part of the neo-Y chromosomes in all studied Thrinchinae species. (Figure 2 and Figure 7). In species with the XX/X0 sex chromosome system the DNA homology was observed only in the X chromosome. We did not identify the autosome that was fused with the ancestral X providing the neo-X and the neo-Y chromosomes.

The C-positive regions in proximal part of the neo-Y chromosomes in all studied Thrinchinae species were enriched with homologous repeats but their number and distribution differed in species of this subfamily (Figure 2 and Figure 7). In *Asiotmethis limbatus*, *Asiotmethis turritus* and *Glyphotmethis efe* strong signal was observed in interstitial C-positive regions of the neo-Y while proximal pericentric C-positive regions showed a weaker signal (Figure 2c–f,m,n and Figure 7). In *Glyphotmethis dimorphus* and *Glyphotmethis holtzi pulchripes* strong signal was observed in pericentric regions and in interstitial C-positive regions of the neo-Y (Figure 2k,l,o,p and Figure 7). Its intensity was similar to the intensity of the signal observed in proximal pericentric C-positive regions of the *A. limbatus*, *A. turritus* and *G. efe* neo-Y chromosomes. Other regions enriched with repetitive DNA homologous to DNA of *AheXl* probe was found in C-negative terminal regions of M_5_, M_7_ and X chromosomes of *A. muricatus* (Figure 2g and Figure 7).

**Figure 8:**
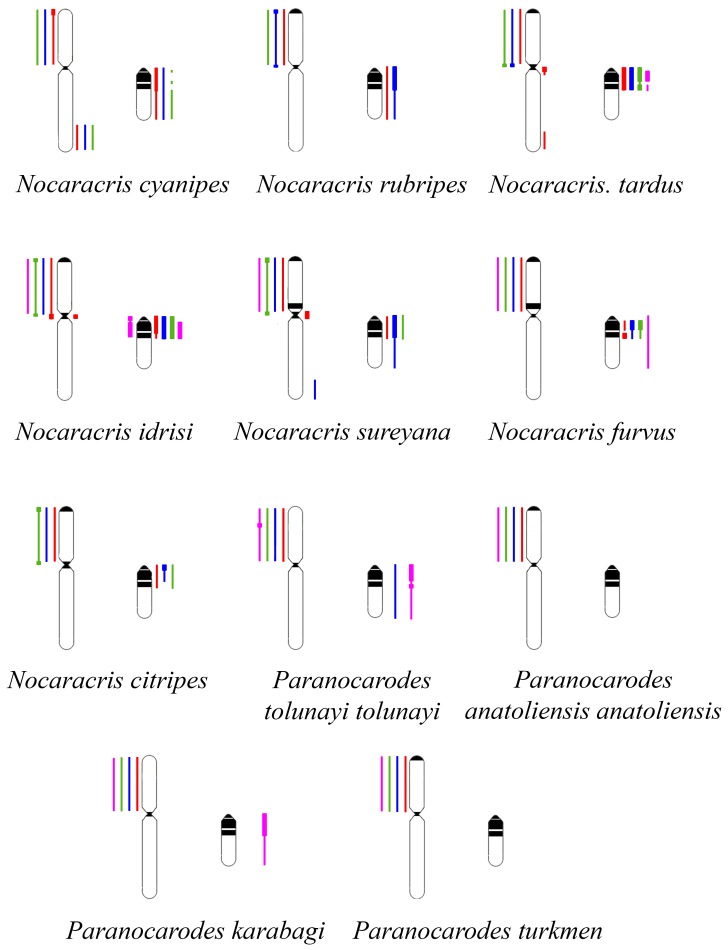
Schematic representation of the neo-sex chromosome painting in Nocarodeini species. The regions painted with the XL DNA probes showed on the left from the chromosome. The regions painted with the neo-Y DNA probes showed on the right from the chromosomes: *NcyXl* and *NcyY* DNA probes (red); *NruXl* and *NruY* DNA probes (blue); *NtaXl* and *NtaY* DNA probes (green); *PtoXl* and *PtoY* DNA probes (cyan). The thick line shows intensive painting, while thin line shows less intensive painting.

The conservatism of the XL was revealed also in the species of Pamphaginae subfamily (Figure 3, Figure 4, Figure 5, Figure 6 and Figure 8). However, homology of C-negative regions in the neo-Y chromosomes and distal part of the XR arm was observed only in closely related species from *Nocaracris* genus (Figure 3, Figure 5a–c,i and Figure 8). Different levels of DNA homology were revealed in C-positive regions of the neo-Y chromosomes in all Nocarodeini species. The *PtoXl* DNA probe painted in addition to the XL also M_7_ and S_8_ autosomes in *Paranocarodes anatoliensis*, *Paranocarodes turkmen* and *Paranocarodes karabagi* (Figure 4 and Figure 6l,p,t).

All DNA probes obtained from chromosomes of Nocarodeini species gave signal in some C-positive regions of autosomes in these species. Number of revealed clusters containing homologous repeats in chromosomes of studied species were in a good agreement with the species position on cladogram showed on the Figure 1. In species of *Nocaracris* genus, more repeat clusters were revealed with “*Nocaracris*” DNA probes than with “*Paranocarodes*” DNA probes (Figure 3 and Figure 4, Supplementary 1–4). Two pairs of DNA probes obtained from *Nocaracris* species (*NcyXl*, *NcyY* and *NtaXl*, *NtaY*) showed DNA variability in C-positive regions of autosomes and sex chromosome in species of this genus (Figure 3a,c,e,g,i,k,q,s,u,w and Figure 4a,c,e,g). The *NruXl* and *NruY* DNA probes painted mainly C-positive regions of the neo-sex chromosomes (Figure 3b,f,j,n,r and Figure 4b,f). However, in *Paranocarodes* species, none of the DNA probes from chromosomes of *Nocaracris* species painted any autosomal C-positive regions (Figure 3d,h,l,t,x and Figure 4d,h).

DNA probes obtained in this study produced different patterns of the painting in different C-positive regions of the neo-Y chromosomes in species of Nocarodeini tribe (Figure 3, Figure 4, Figure 5, Figure 6 and Figure 8). In general, “*Nocaracris*” DNA probes hybridized more intensively with C-positive regions of neo-Y chromosomes of Nocaracris species and significantly less intensive with the neo-Y chromosomes of Paranocarodes species. The *PtoY* DNA probe derived from *P. tolunayi* painted C-positive regions of the neo-Y chromosome in *Nocaracris furvus*, *N. tardus*, *Nocaracris idrisi* and *P. karabagi* (Figure 3l,p,x, Figure 4h,t, Figure 5l,p,x, Figure 6h,t and Figure 8). In other species of Nocarodeini tribe it painted C-positive regions only slightly. The unusual result was observed in cross-hybridization of the *NtaY* and *PtoY* DNA probes with chromosomes of *P. tolunayi* and *N. tardus*, respectively. The *NtaY* DNA probe showed low level of homology with C-positive region of neo-Y chromosome of *P. tolunayi*, while the *PtoY* DNA probe revealed intensive signal in C-blocks of the neo-Y chromosome in *N. tardus* (Figure 3l,o).

## 4. Discussion

### 4.1. Particularities of Comparative Cytogenetics in Grasshoppers

The Pamphagidae family is one of the few taxa among grasshoppers, which includes phylogenetic lineages characterized by the neo-sex chromosome systems. Recently species of this family were karyotyped and the neo-sex chromosomes were described with C-banding of mitotic and meiotic chromosomes and FISH of 28S rDNA and telomeric repeats [23,24,32,33]. However, understanding of the neo-sex chromosome evolution requires identification of the autosomes fused with the ancestral X, description of their degradation process and characterization of newly arisen clusters of DNA repeats. Generation of microdissected DNA libraries followed by FISH on chromosomes of close related species and sequencing of these libraries look like promising approach. Unfortunately, the large size of grasshopper genomes saturated with repetitive DNA hinders the application of these modern molecular cytogenetic techniques. The grasshoppers’ genomes are the largest genomes among insects and vary from 3.76 Gb to 16.56 Gb [34]. Up to date the studies of grasshopper chromosomes were mainly restricted to studies of C-positive regions and repeat clusters performed with classical analysis of chromosome morphology, C-banding and FISH with different repeats [35,36,37,38,39,40]. Cytogenetic studies with the application of microdissected DNA libraries and probes followed by FISH and DNA sequencing were also devoted to the study of repetitive DNA, mainly its distribution in C-positive regions of the A and B chromosomes [26,41,42,43,44]. The whole genome sequencing and assembly were performed only for *Locusta migratoria* [45]. According to data of this genome sequencing, approximately 60% of its genome are repetitive DNA. However, the top 10 repetitive DNA families represent only 10% of the whole genome [45]. A high level of repetitive DNA was also shown by the low coverage sequencing of the genome of *Schistocerca gregaria* [46]. These data suggest that acridid genomes are significantly enriched with complex repeats of different kinds. Recently the generation of microdissected DNA probes from C-negative B chromosomes of *Abracris flavolineata* and subsequent FISH of this probe with chromosomes of this species revealed intensive hybridization signal in particular C-negative regions of B chromosome, X chromosome and some autosomes. It was suggested that these signals were located in C-negative regions enriched with repetitive DNA of unidentified nature [44]. As a result and in contrast to mammalian chromosomes [47,48,49], for a long time whole chromosome probe generation and Chromosome *in situ* suppression (CISS)-hybridization on grasshopper chromosomes provided no data on homology of C-negative regions even in close related species.

Development of the VISSIS analysis [30] improved the identification of the FISH signal produced by unique and repetitive chromosome specific DNA sequences after FISH of microdissected DNA probes. The application of the VISSIS analysis allowed successfully using this approach for the study of neo-sex chromosomes in Pamphagidae grasshoppers. We should note that in C-positive regions the specific FISH signal was derived obviously from the chromosome specific repetitive DNA. The interpretation of the specific FISH signal in C-negative regions is more complicated. The total FISH signal is the sum of unique and repetitive DNA. The VISSIS analysis allows eliminating the FISH signal derived from the repetitive DNA present in the both DNA probes. If one of the microdissected chromosomes contained the dispersed chromosome specific repeats, after VISSIS analysis its C-negative regions would show the signal derived from both, unique and chromosome specific dispersed repetitive DNA. Furthermore, if a chromosome contains only chromosome specific dispersed repeats the VISSIS analysis will also reveal chromosome specific FISH signal [30]. We cannot exclude the existence of chromosome specific dispersed repeats or the enrichment of the sex chromosomes of grasshoppers with such specific dispersed repeats. Furthermore, the intensive painting of M_7_ and S_8_ autosomes in *P. anatoliensis*, *P. turkmen* and *P. karabagi* with *PtoXl* probe provides indirect evidence of the presence of the chromosome specific dispersed repeats in these chromosomes. Other DNA probes that painted the XLs in Paranocarodes species did not paint these autosomes, suggesting the absence of homology of the M_7_ and S_8_ autosomes in these species to the regions of the XL of *P. tolunayi*. On the other hand, the XLs in *N. cyanipes*, *N. rubripes* and *N. tardus* were painted with *PtoXl* probe suggesting that hybridization signal does not come from these dispersed repeats. Additional studies are required for the estimation of the significance of the chromosome specific dispersed repeats for the identification of the homologous chromosomes and the chromosome regions in grasshoppers.

### 4.2. Comparative Cytogenetics of C-Negative Regions in the Pamphagidae Neo-Sex Chromosomes

The complex approach of the chromosome painting and the VISSIS analysis revealed the homologous C-negative regions in chromosomes of Pamphagidae grasshoppers. This approach showed a high level of homology of the XLs and the X chromosomes within Thrinchinae subfamily (Figure 7) and the XLs in the species of Nocarodeini tribe (Figure 8). These data are in a good agreement with the classical hypothesis on karyotype conservatism in grasshoppers [5]. However, this is the first direct evidence of the X chromosome conservatism at least in closely related species. The performed painting also revealed the homology between the proximal parts of the neo-Ys in different Thrinchinae species supporting the hypothesis of the monophyletic neo-Y chromosome origin in this lineage. Patterns of painting in the species of Nocarodeini tribe also support the hypothesis of the monophyletic origin of the neo-Ys within this group.

Unfortunately, the determination of homologous regions in chromosomes of species from the different subfamilies was unsuccessful due to accumulation of DNA divergence between these groups in the Pamphagidae family. Therefore, we cannot exclude that neo-sex chromosomes in different phylogenetic linages of Pamphagidae family are results of the X chromosome fusion with different autosomes.

We observed decreased painting intensity of chromosomes in species with XX/X0 sex chromosome system using *AheXl* and *AheYcen* DNA probes. Based on the well-known rule that the longer time of divergence between species leads to the accumulation of differences in their DNA, we suppose that in Thrinchinae subfamily species with XX/X0 sex chromosome system diverged from the group of the species the with neo-sex chromosomes a relatively long time ago. FISH with *AheXl* and *AheYcen* DNA probes provided in C-negative regions of the X chromosomes in *A. muricatus* and *G. adaliae* less intensive painting than on the XL in species with neo-sex chromosomes.

Probably, further investigations including Next Generation Sequencing techniques of microdissected libraries and bioinformatic analysis using the draft of *Locusta migratoria* genome as a referent genome [50,51] can determine the homology of chromosome regions in more distant species of grasshoppers.

### 4.3. Comparative Cytogenetics of C-Positive Regions of Chromosomes in Pamphagidae Grasshoppers

Evolution of the neo-Y chromosomes in Pamphagidae grasshoppers is associated with the formation of new C-positive regions and the elimination of C-negative regions located between them. It makes actual the comparison of C-positive regions of autosome with C-positive regions of neo-sex chromosomes. Previously it was shown that DNA of C-positive regions can share no homology even in closely related species suggesting its rapid evolution [52,53,54]. We observed similar diversity of the DNA composition between C-positive regions of chromosomes in current work for many studied species. (“the DNA composition” we use here and below to indicate the special set of DNA elements present in the region without any information of their sequences) Furthermore, C-positive regions could have different DNA composition in different chromosomes of the one species or the same chromosomes in different populations belonging to the one species (unpublished data). These data suggest the rapid DNA evolution in C-positive regions. One of the possible mechanisms was proposed by Fry and Salser [55] in the “library hypothesis.” According to this hypothesis, in C-positive regions different types of satellite repeats are present, however due to differential amplification, repeats of one family are present as a major fraction while repeats of other families present as a minor fraction or even in a few copies. Even in closely related species, the independent amplification of repeats from different families can lead to the formation of C-positive regions enriched with different repeats [53]. In our study, we registered the presence of homologous DNA in pericentric regions of chromosomes in a group of closely related species. We suppose that these unusual results might indicate phylogenetic proximity of the species.

Small pericentric C-positive regions of the neo-Y chromosomes also showed high diversity of DNA composition whereas interstitial C-positive regions in the proximal part of the neo-Ys showed a higher level of homology in each of phylogenetic lineages (Figure 2, Figure 3, Figure 4, Figure 7 and Figure 8). In species of Thrinchinae lineage, interstitial C-positive regions of neo-Ys contained homologous DNA in all species of *Asiotmethis* and *Glyphotmethis* genera (Figure 2 and Figure 7). We should remind that these species are very closely related and in earlier taxonomy they belonged to the one genus [56]. We suggest that these patterns of the C-positive region in the neo-Ys are caused by a series of paracentric inversions that transferred repeats from proximal C-block to a more distal location. Amplification of transferred repeats, formation of new interstitial C-blocks and elimination of the C-negative regions located between them lead to the Y chromosome degradation associated with the loss of C-negative regions and enlargement of proximal C-positive region. We observed advanced stages of this process in the neo-Ys of Nocarodeini tribe. They consist of several large proximal C-positive and decreased in size distal C-negative regions. Taken together, C-positive regions were approximately a half of the XR [23,24].

Observed homology of repeats in interstitial C-positive regions of the Ys in each lineage is of special interest. Probably the repeats have to maintain the mobility and the ability to induce chromosome rearrangements to cause Y chromosome degradation. That leads to amplification and distribution of the repeats possessing these properties and belonging probably to the same family. In different lineages, repeats from different families could be involved in this process. As a result, DNA of interstitial C-positive regions in the neo-Ys of different species can contain different repeats. We observed this phenomenon in species from Nocarodeini tribe. It is possible that there are no specific repeats involved in the Y chromosome degradation but all of them should possess special properties for translocation and amplification. The sequencing of microdissected DNA libraries derived from these C-positive regions can answer the question on the DNA content of these repeats.

Most likely, C-positive regions in the neo-Ys consist of the mixture of repeats. The repeats of the major fraction should possess the properties discussed above. If different repeats from this mixture possess these properties, the scenario described in the “library hypothesis” [55] can also be applied for C-positive regions in the neo-Ys. Asymmetric results of cross-hybridization with *NtaY* and *PtoY* DNA probes on the neo-Ys in *N. tardus* and *P. tolunayi* are in a good agreement with this hypothesis. We believe that the major fraction of repetitive DNA in C-positive region of the neo-Y in *P. tolunayi* is a minor fraction of repetitive DNA in the C-positive region of the neo-Y in *N. tardus*. Recently it was shown that tandem satellite repeats are accumulated in the neo-Y chromosome of *Eneoptera surinamensis* [57]. This type of repeats may also be abundant in neo-Y chromosomes in Pamphagidae grasshoppers.

### 4.4. Conjugation of the Neo-Sex Chromosomes in Pamphagidae Species

In Pamphagidae species, the neo-Y is derived from an autosome and we should not expect restriction of the recombination between the neo-Y and XR on the initial stage of its evolution. In Thrinchini species a large region of the neo-Y conjugated with the XR with formation of one or two chiasmata [24]. Nevertheless, the presence of interstitial C-positive regions in the proximal part of the neo-Y and no C-positive regions in the corresponding part of the XR verifies the hypothesis that suppression of recombination took place in the proximal part of the Y. This suppression could facilitate the process of the neo-Y degradation. On the further stages of the heteromorphization, the suppression of recombination could be self-enhancing and spread to the distal part of the neo-Y chromosome accompanied by the accumulation of repetitive sequences and changing in chromatin stricture. In the future, analysis of recombination nodule distributions along the pachytene chromosomes with immunostaining of synaptonemal complex and MLH1 proteins in the Thrinchini species can show the suppression of recombination and help with the estimation of its role in the neo-sex chromosome evolution in Pamphagidae.

## 5. Conclusions

Pamphagidae grasshoppers represent a unique group with the neo-sex chromosomes present at the different stages of the heteromorphisation. Two different lineages of the neo-sex chromosomes in this group exhibit similar pathways of the neo-Y degradation. These pathways include expansion and amplification of different repeats started in the proximal chromosome region and spreading in the distal direction. Progressive elimination of the C-negative region located between newly arisen C-blocks leads to the formation of the large proximal C-positive region. This degradation was probably facilitated by the suppression of recombination in the proximal region of the neo-Ys. Further studies of the neo-sex chromosome evolution in Pamphagidae grasshoppers and the neo-sex chromosome behavior in meiosis can discover the mechanisms of the Y chromosome degradation in more details and broaden our knowledge of the sex-chromosome evolution in general.

## Figures and Tables

**Table 1:** List of species studied and collection places.

Taxa	Species *	Location	Specimen Number
Thrinchini, Thrinchinae	*Asiotmethis muricatus* (Pallas, 1771)	Kazakhstan	12
		50°45.377′ N; 51°37.493′ E	
	*Asiotmethis turritus* (Fischer von Waldheim, 1833)	Armenia	9
		40°10.220′ N; 44°.22.458′ E	
	*Asiotmethis heptapotamicus songoricus* (Shumakov, 1949)	Kazakhstan	19
		47°52.494′ N; 80°6.435′ E	
	*Asiotmethis limbatus* (Charpentier, 1845)	Bulgaria, Harmanli **	11
	*Glyphotmethis adaliae* (Uvarov, 1928)	Turkey	6
		37°37.518′ N; 29°13.948′ E	
	*Glyphotmethis dimorphus dimorphus* (Uvarov, 1934)	Turkey	11
		38°18.438′ N; 31°43.676′ E	
	*Glyphotmethis efe* (Ünal, 2007)	Turkey	10
		39°03.285′ N; 29°26.741′ E	
	*Glyphotmethis holtzi pulchripes* (Uvarov, 1943)	Turkey	14
		38°46.688′ N; 34°51.215′ E	
Pamphaginae, Nocarodeini	*Nocaracris citripes* (*syn. Paranocaracris citripes citripes* (Uvarov, 1949))	Turkey	13
		37°05.779′ N; 28°50.972′ E	
	*Nocaracris cyanipes* (Fischer von Waldheim, 1846)	Armenia	9
		40°39.116′ N; 44°58.525′ E	
	*Nocaracris rubripes* (*syn. Paranocaracris rubripes* (Fischer von Waldheim, 1846))	Armenia	3
		40°23.111′ N; 44°15.324′ E	
	*Nocaracris furvus furvus* (*syn. Oronothrotes furvus* (Mishchenko, 1951))	Turkey	27
		38°21.258′ N; 28°06.713′ E	
	*Nocaracris idrisi* (*syn. Paranocaracris citripes idrisi* (Karabağ, 1953))	Turkey	9
		40°35.385′ N; 31°17.293′ E	
	*Nocaracris sureyana* (*syn. Paranocaracris sureyana* (Ramme, 1951))	Turkey	3
		39°02.353′ N; 29°17.074′ E	
	*Nocaracris tardus* Ünal et al. 2016 (*syn.* *Paranocaracris* sp.)	Turkey	7
		38°16.672′ N; 31°19.491′ E	
	*Paranocarodes anatoliensis anatoliensis* (*syn. Paranocarodes fieberi anatoliensis* Demirsoy, 1973)	Turkey	2
		37°48.527′ N; 30°45.472′ E	
	*Paranocarodes karabagi* (*syn. Pseudosavalania karabagi* Demirsoy, 1973)	Turkey	15
		39°03.285′ N; 29°26.741′ E	
	*Paranocarodes tolunayi tolunayi* (*syn. Paranocarodes fieberi tolunayi* Karabag, 1949)	Turkey	2
		40°40.937′ N; 31°46.489′ E	
	*Paranocarodes turkmen* (Ünal, 2014)	Turkey	2
		39°54.453′ N; 30°41.477′ E	

* The current species names were used while synonyms according to old nomenclature were place in brackets; ** Specimens were collected in summer of 1994, 1995 and no GPS coordinates are available.

## Supplementary Materials

The following are available online at www.mdpi.com/2073-4425/8/11/323/s1. Figure S1: Scheme of chromosome painting in Nocarodeini species with *NcyXl* and *NcyY* DNA probes, Figure S2: Scheme of chromosome painting in Nocarodeini species with *NruXl* and *NruY* DNA probes, Figure S3: Scheme of chromosome painting in Nocarodeini species with *NtaXl* and *NtaY* DNA probes, Figure S4: Scheme of chromosome painting in Nocarodeini species with *PtoXl* and *PtoY* DNA probes, Figure S5: Definition of intensity levels of hybridization signals on the example of *N. cyanipes* chromosome painting with *NcyXl* and *NcyY* DNA probes, Figure S6: Definition of intensity levels of hybridization signals on the example of *N. cyanipes* chromosome painting with *NcyXl* and *NcyY* DNA probes after VISSIS analysis.
