# Supplementary material for: Origin and Evolution of the Neo-Sex Chromosomes in Pamphagidae Grasshoppers through Chromosome Fusion and Following Heteromorphization
Source: Genes (Basel). 2017 Nov 13;8(11):323. doi: 10.3390/genes8110323 (PMC5704236; doi:10.3390/genes8110323)
Supplement: Supplementary file 1 [file genes-08-00323-s001.zip › Supplementary.docx]

Supplementary to the article “Origin and evolution of the neo-sex chromosomes in Pamphagidae grasshoppers through chromosome fusion and their following heteromorphization”

Ilyas Yerkinovich Jetybayev ^1,2,^*, Alexander Gennadievich Bugrov ^2,3^ Olesya Georgievna Buleu^2,3^, Anton Gennadievich Bogomolov^1,3^ and Nikolay Borisovich Rubtsov^1,3^

^1^ The Federal Research Center Institute of Cytology and Genetics, Russian Academy of Sciences, Siberian Branch, Lavrentjev Ave., 10, 630090, Novosibirsk, Russia

^2^ Institute of Systematics and Ecology of Animals, Russian Academy of Sciences, Siberian Branch, Frunze str. 11, 630091 Novosibirsk, Russia

^3^ Novosibirsk State University, Pirogov str., 2, 630090, Novosibirsk, Russia,

***** Correspondence: jetybayev@mail.ru; Tel.: +7-383-363-49-63*1027


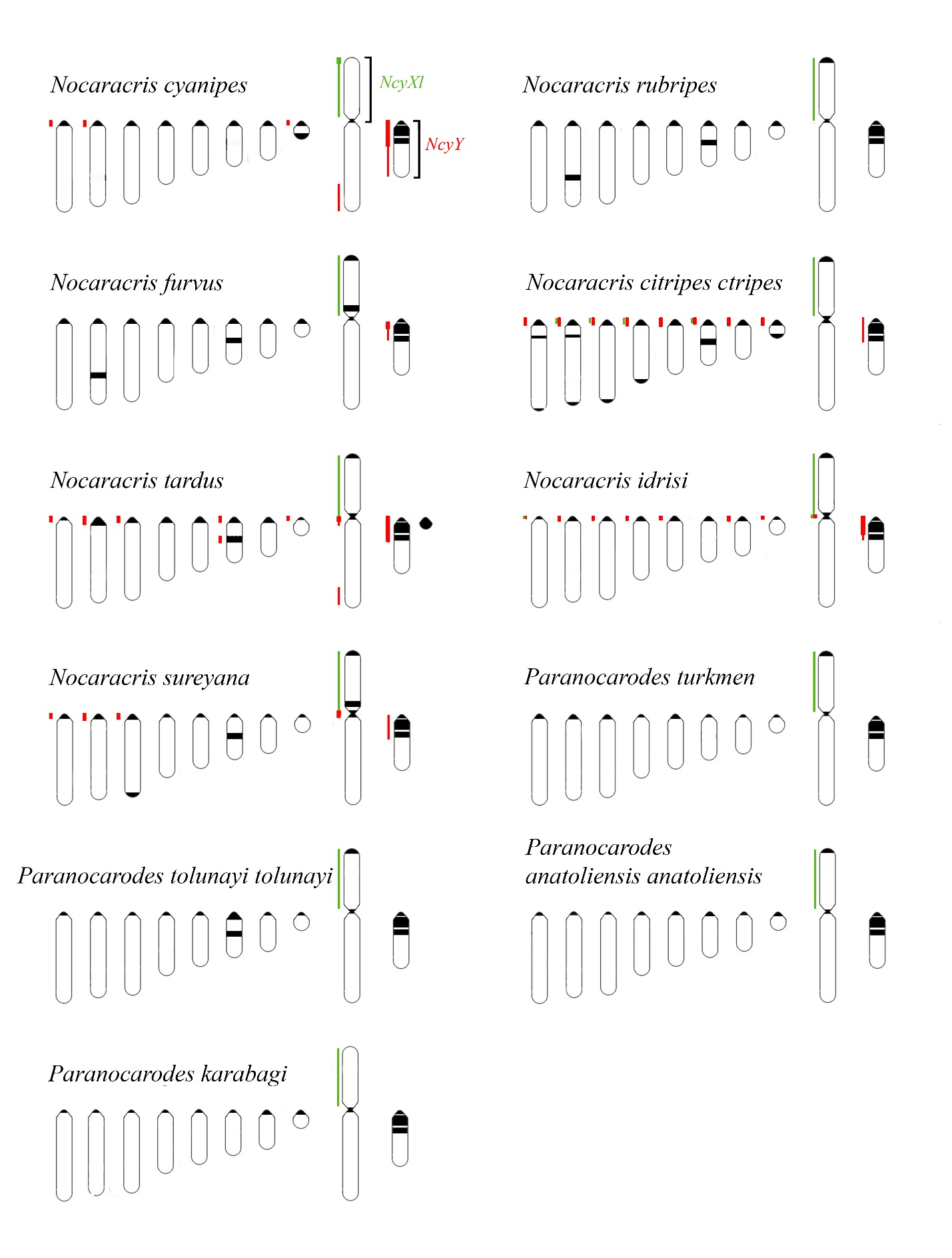


**Figure S2**: Scheme of chromosome painting in Nocarodeini species with *NcyXl* and *NcyY* DNA probes. The thick line shows intensive painting, while thin line shows less intensive painting. Black brackets indicate regions of microdissection. Distribution of C-positive regions on ideograms (black) is shown according to described earlier [1,2]

**
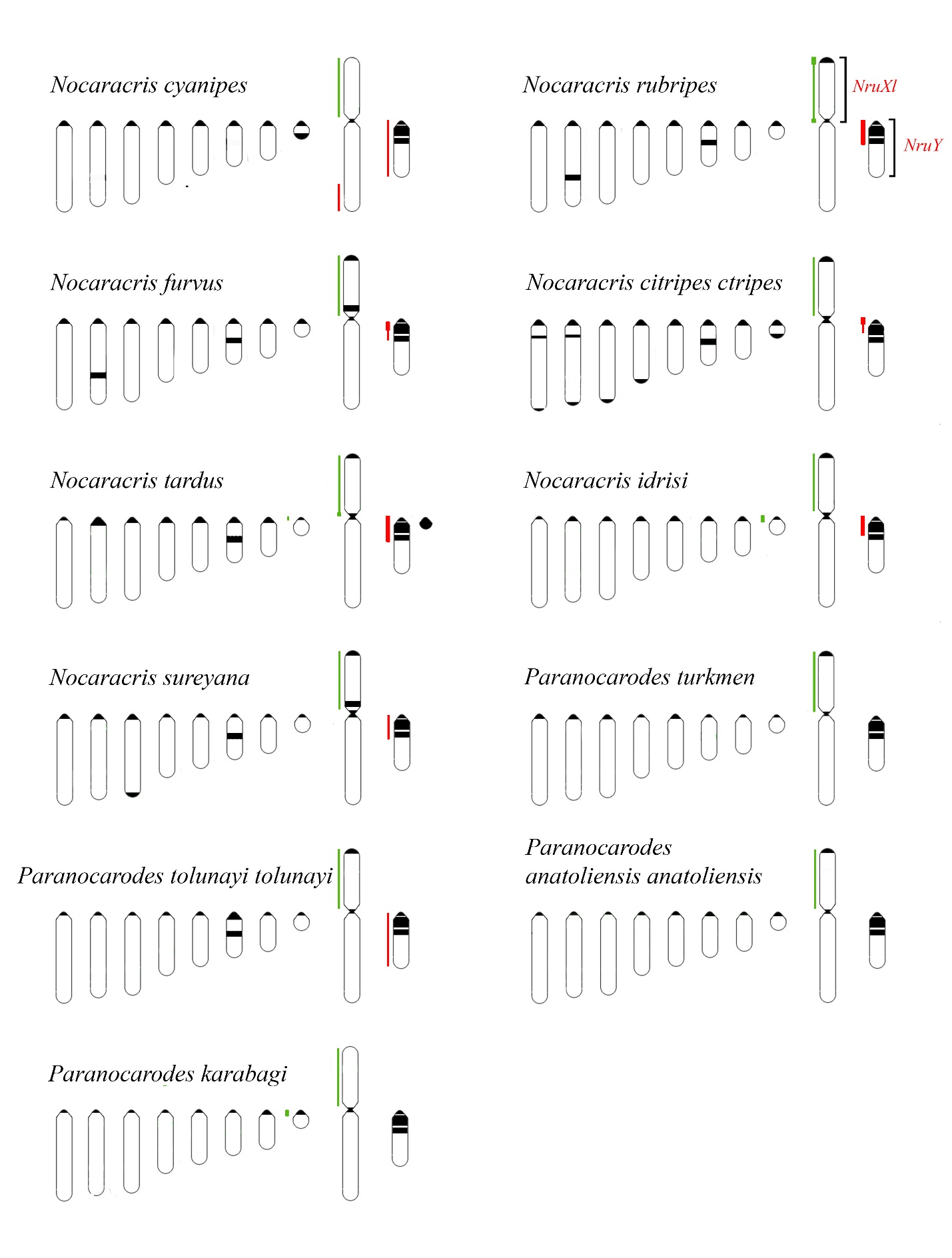
Figure S3** Scheme of chromosome painting in Nocarodeini species with *NruXl* and *NruY* DNA probes. The thick line shows intensive painting, while thin line shows less intensive painting. Black brackets indicate regions of microdissection. Distribution of C-positive regions on ideograms (black) is shown according to described earlier [1,2]

**
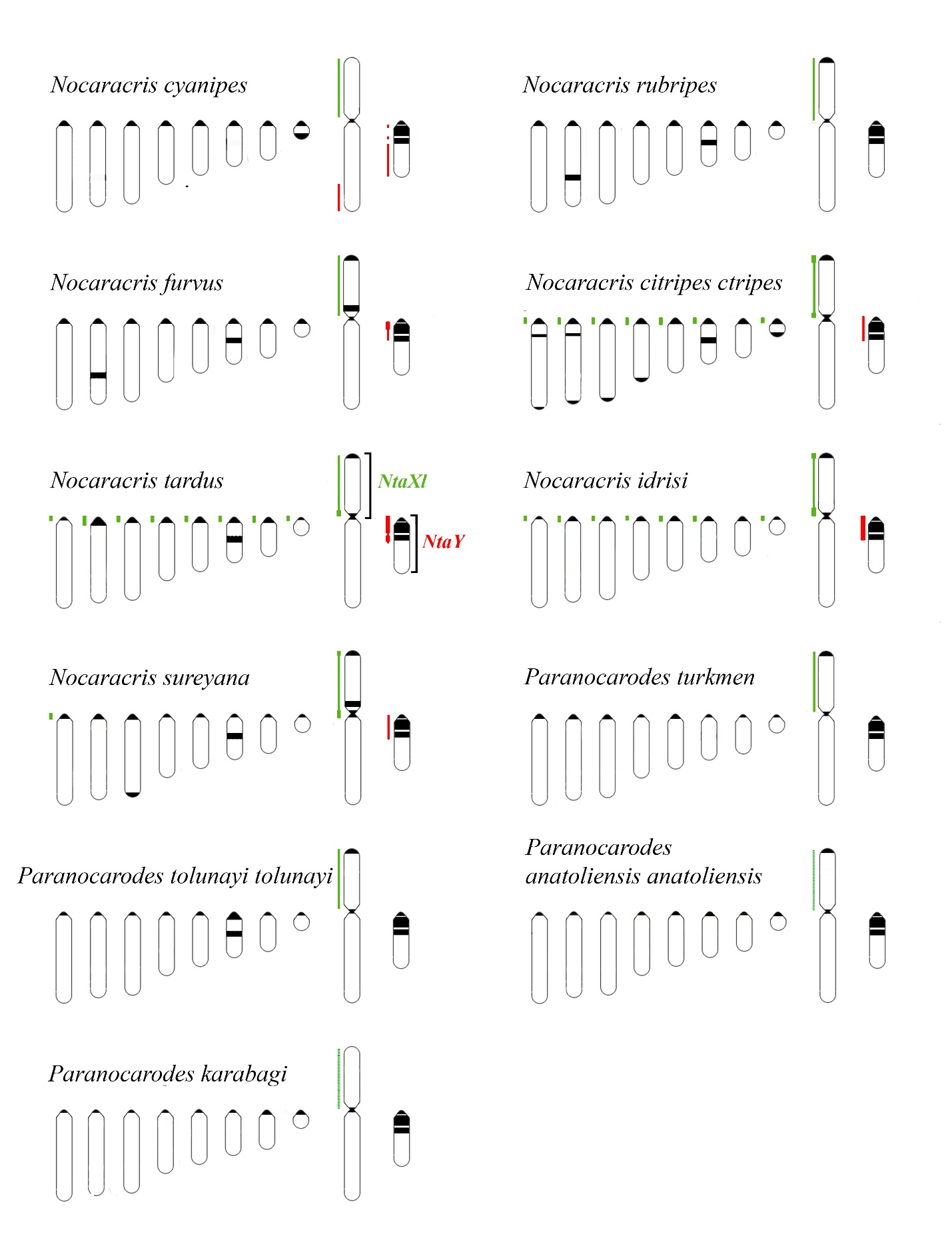
Figure S4** Scheme of chromosome painting in Nocarodeini species with *NtaXl* and *NtaY* DNA probes. The thick line shows intensive painting, while thin line shows less intensive painting. Black brackets indicate regions of microdissection. Distribution of C-positive regions on ideograms (black) is shown according to described earlier [1,2]

**
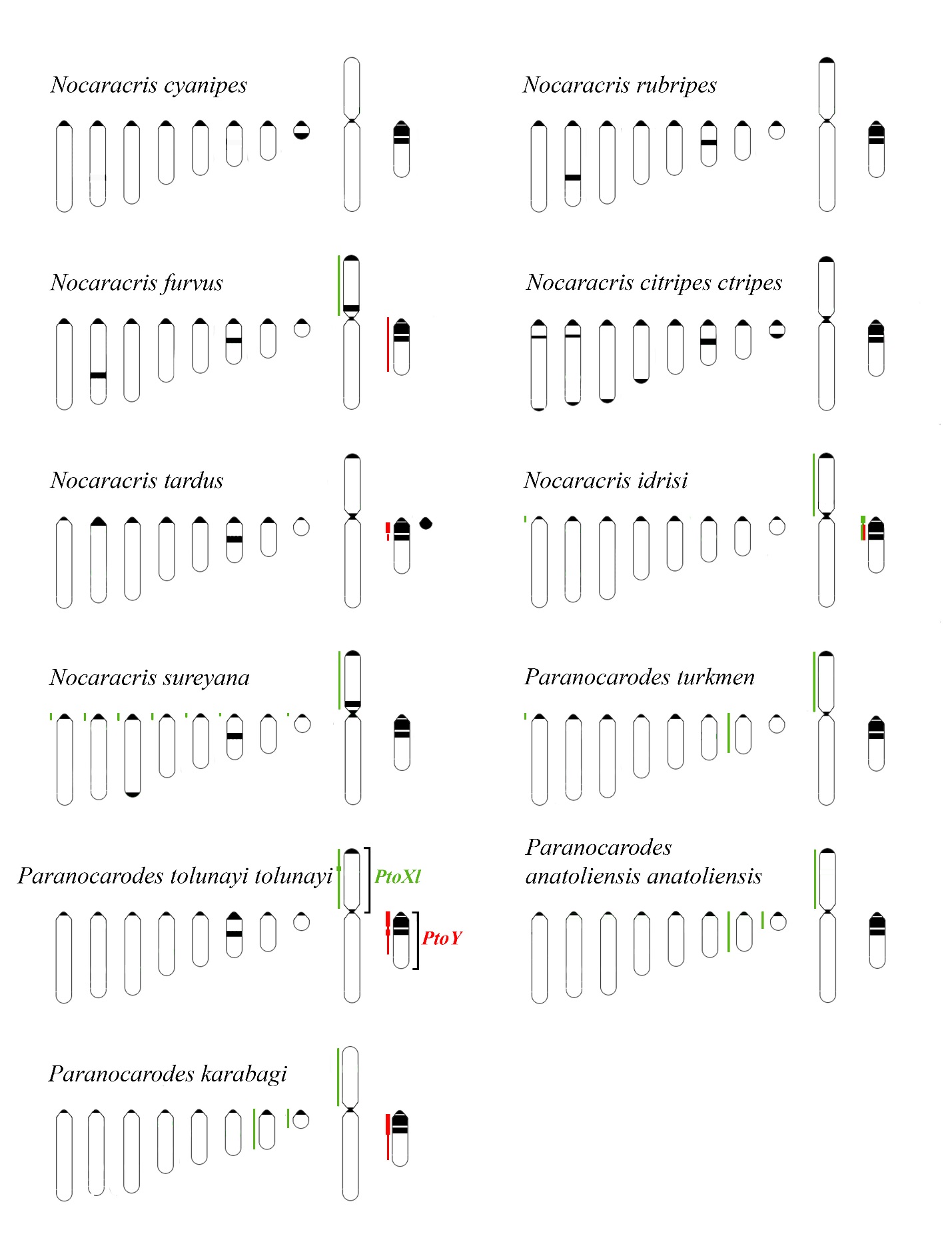
Figure S5** Scheme of chromosome painting in Nocarodeini species with *PtoXl* and *PtoY* DNA probes. The thick line shows intensive painting, while thin line shows less intensive painting. Black brackets indicate regions of microdissection. Distribution of C-positive regions on ideograms (black) is shown according to described earlier [1,2]


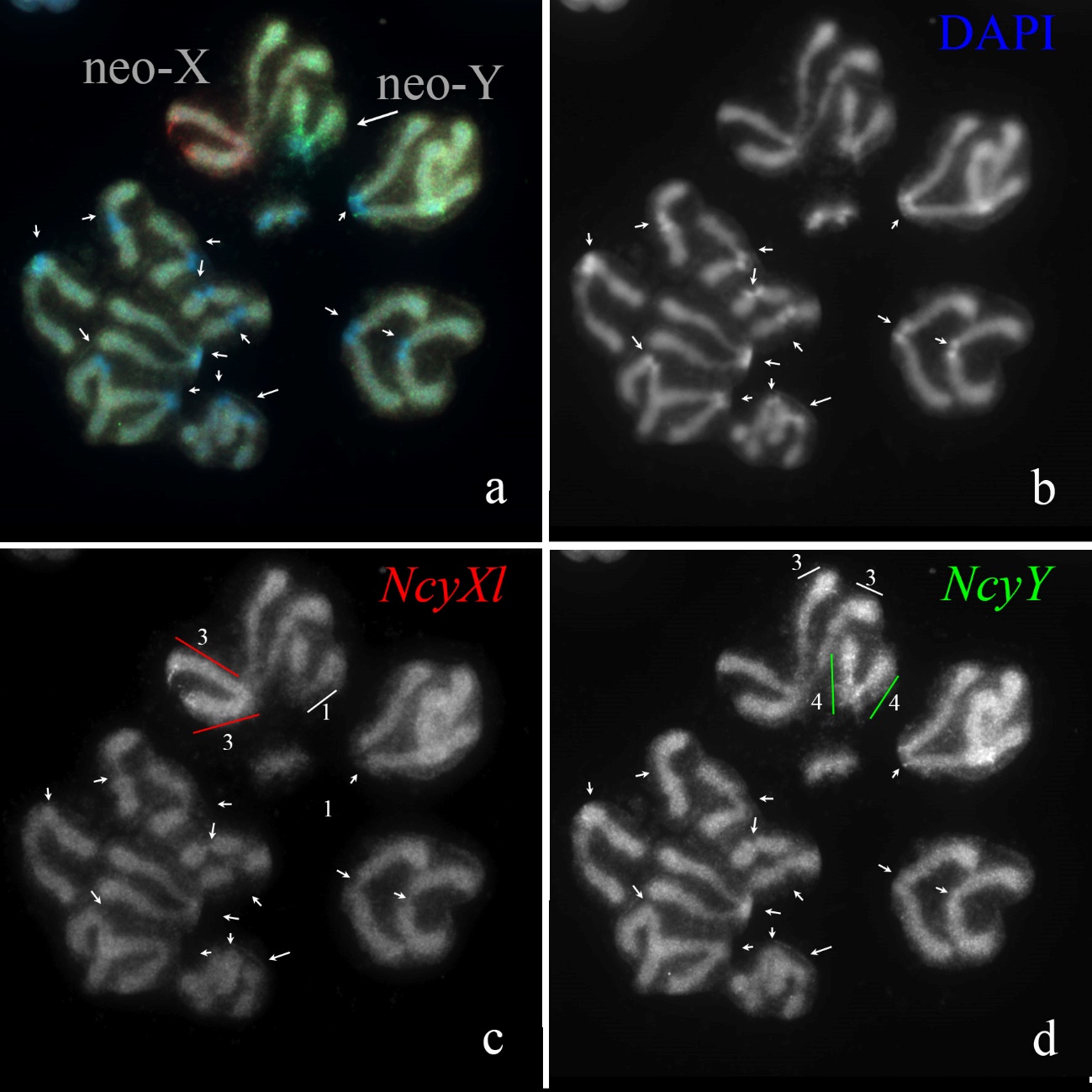


**Figure S5**: Definition of intensity levels of hybridization signals on the example of *N. cyanipes* chromosome painting with *NcyXl* and *NcyY* DNA probes: (a) merged image; (b) DAPI staining; (c) *NcyXl* DNA probe painting; (d) *NcyY* DNA probe painting. Lines and arrows indicate regions characterized with different four types of intensity:

1. FISH signal of background intensity observed in C-positive regions of some autosomes containing no interspersed repeats (*NcyX* probe didn’t painted C-blocks arrows and white line on (c).);
2. FISH signal produced by interspersed repeats in C-negative regions of the autosomes (most of the C-negative regions);
3. Specific FISH signal in C-negative regions of chromosome regions after reverse painting (XL arm painted by *NcyXl* probe (red line) and distal region of XR arm (white line on (d)));
4. Strong specific FISH signal in C-positive regions containing repeats homologous to repeats of dissected neo-Y chromosome after reverse painting (C-positive regions of neo-Y(green line)).

**
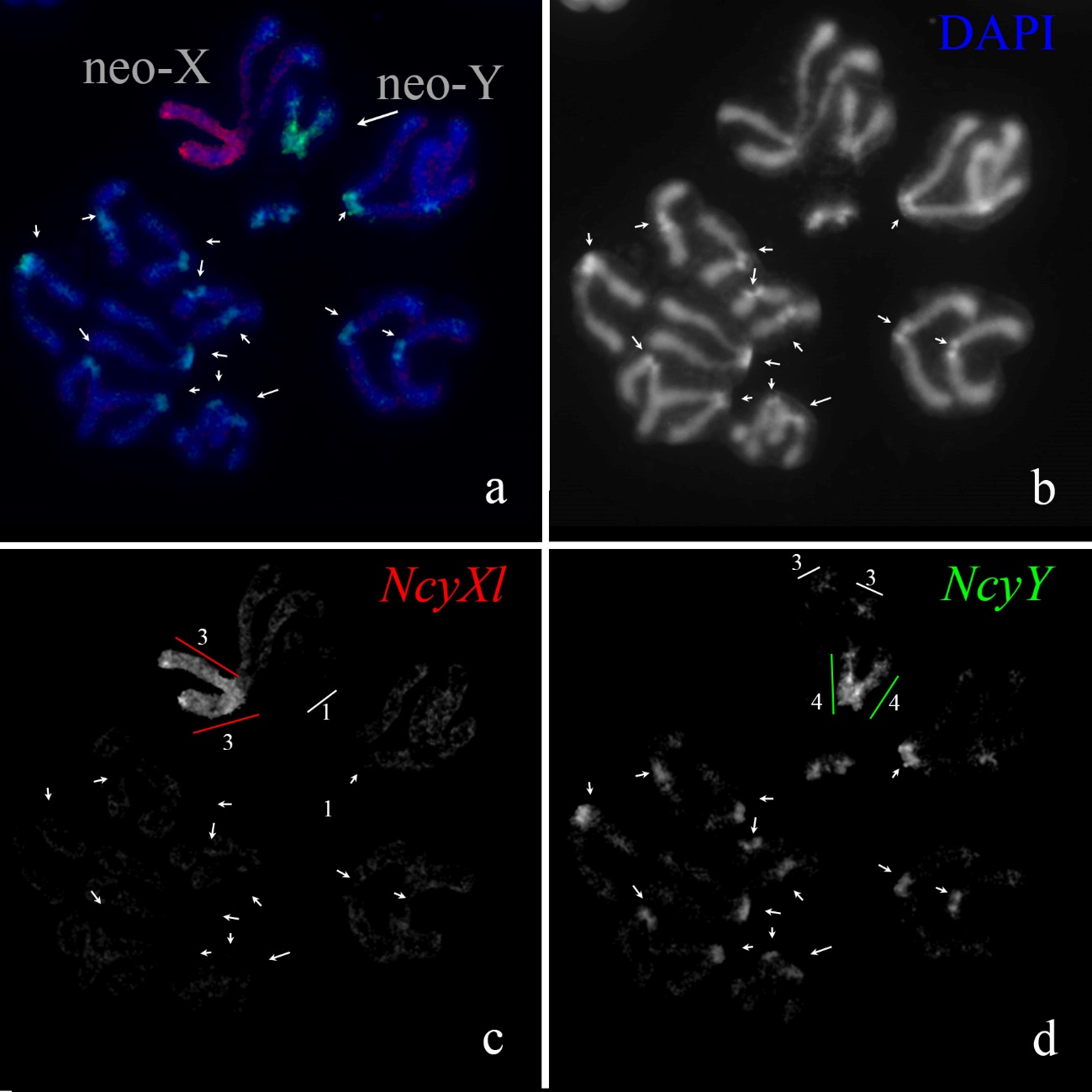
**

**Figure S6**: Definition of intensity levels of hybridization signals on the example of *N. cyanipes* chromosome painting with *NcyXl* and *NcyY* DNA probes after VISSIS analysis: (a) merged image; (b) DAPI staining; (c) *NcyXl* DNA probe painting; (d) *NcyY* DNA probe painting. Lines and arrows indicate regions characterized with different four types of intensity:

1. FISH signal of background intensity observed in C-positive regions of some autosomes containing no interspersed repeats. Signal was suppressed by VISSIS;
2. FISH signal produced by interspersed repeats in C-negative regions of the autosomes. Signal was suppressed by VISSIS;
3. Specific FISH signal in C-negative regions of chromosome regions after reverse painting. Signal was enhanced by VISSIS;
4. Strong specific FISH signal in C-positive regions containing repeats homologous to repeats of dissected neo-Y chromosome after reverse painting Signal was enhanced by VISSIS.

was suppressed by VISSIS analysis.

**Literature**

1. Bugrov, A. G.; Jetybayev, I. E.; Karagyan, G. H.; Rubtsov, N. B. Cytogenetics Sex chromosome diversity in Armenian toad grasshoppers ( Orthoptera , Acridoidea , Pamphagidae ). *Comp. Cytogenet.* **2016**, *10*, 45–59, doi:10.3897/CompCytogen.v10i1.6407.

2. Jetybayev, I. Y.; Bugrov, A. G.; Ünal, M.; Buleu, O. G.; Rubtsov, N. B. Molecular cytogenetic analysis reveals the existence of two independent neo-XY sex chromosome systems in Anatolian Pamphagidae grasshoppers. *BMC Evol. Biol.* **2017**, *17*, 20, doi:10.1186/s12862-016-0868-9.
