# Supplementary figures and images for: Origin and Evolution of the Neo-Sex Chromosomes in Pamphagidae Grasshoppers through Chromosome Fusion and Following Heteromorphization
Source: Genes (Basel). 2017 Nov 13;8(11):323. doi: 10.3390/genes8110323 (PMC5704236; doi:10.3390/genes8110323)

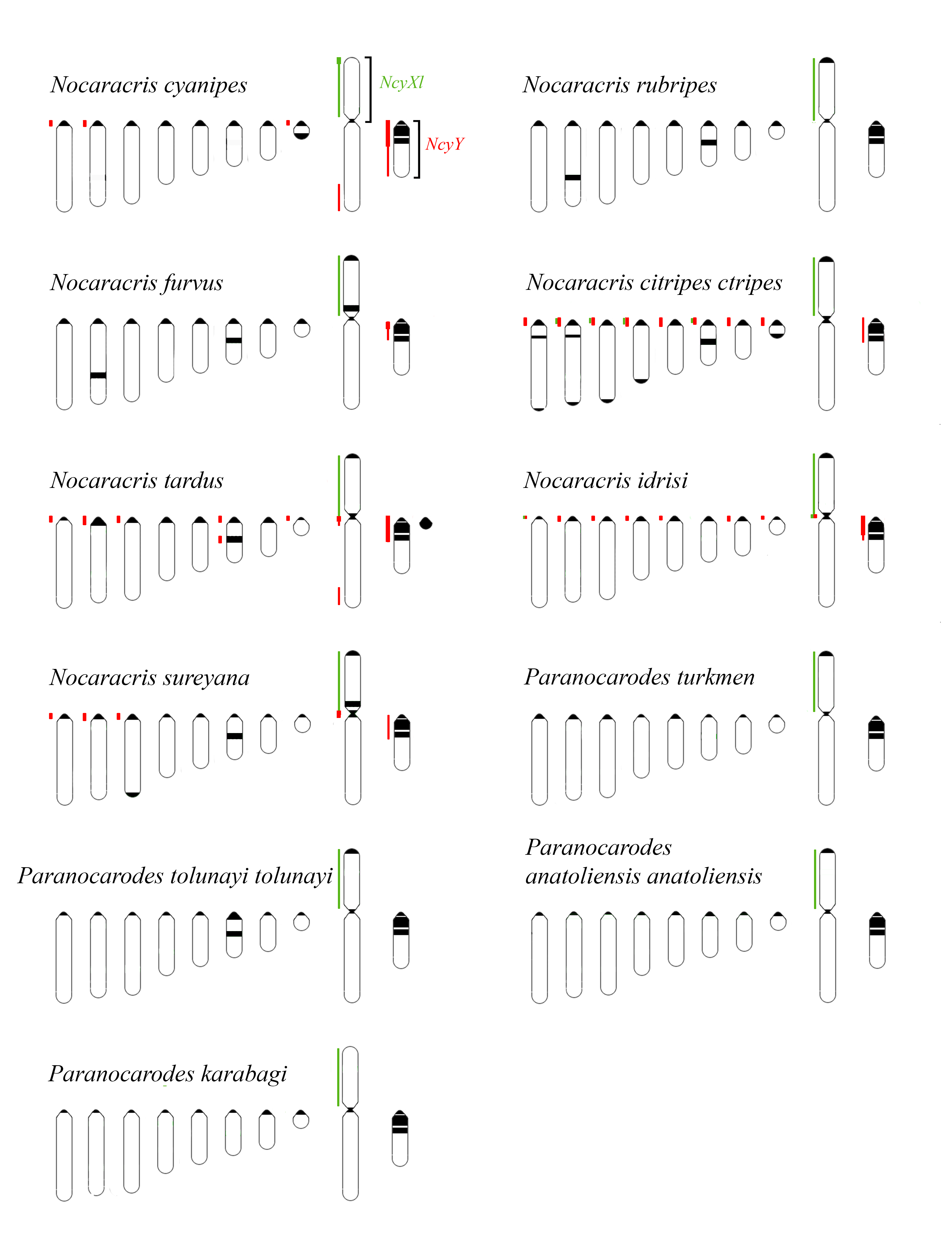

Supplement: Supplementary file 1 [file genes-08-00323-s001.zip › Fig S1.tif]

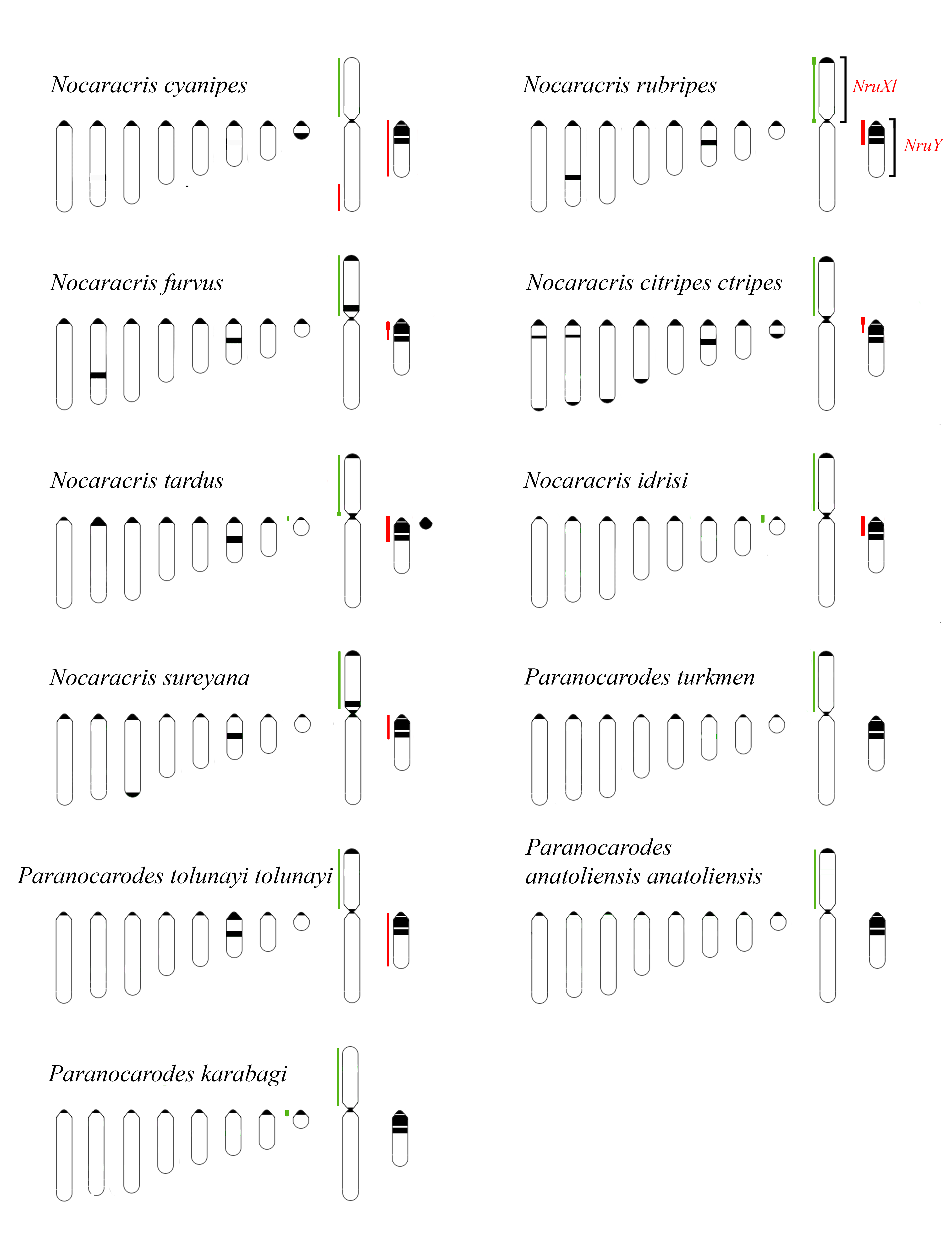

Supplement: Supplementary file 1 [file genes-08-00323-s001.zip › Fig S2.tif]

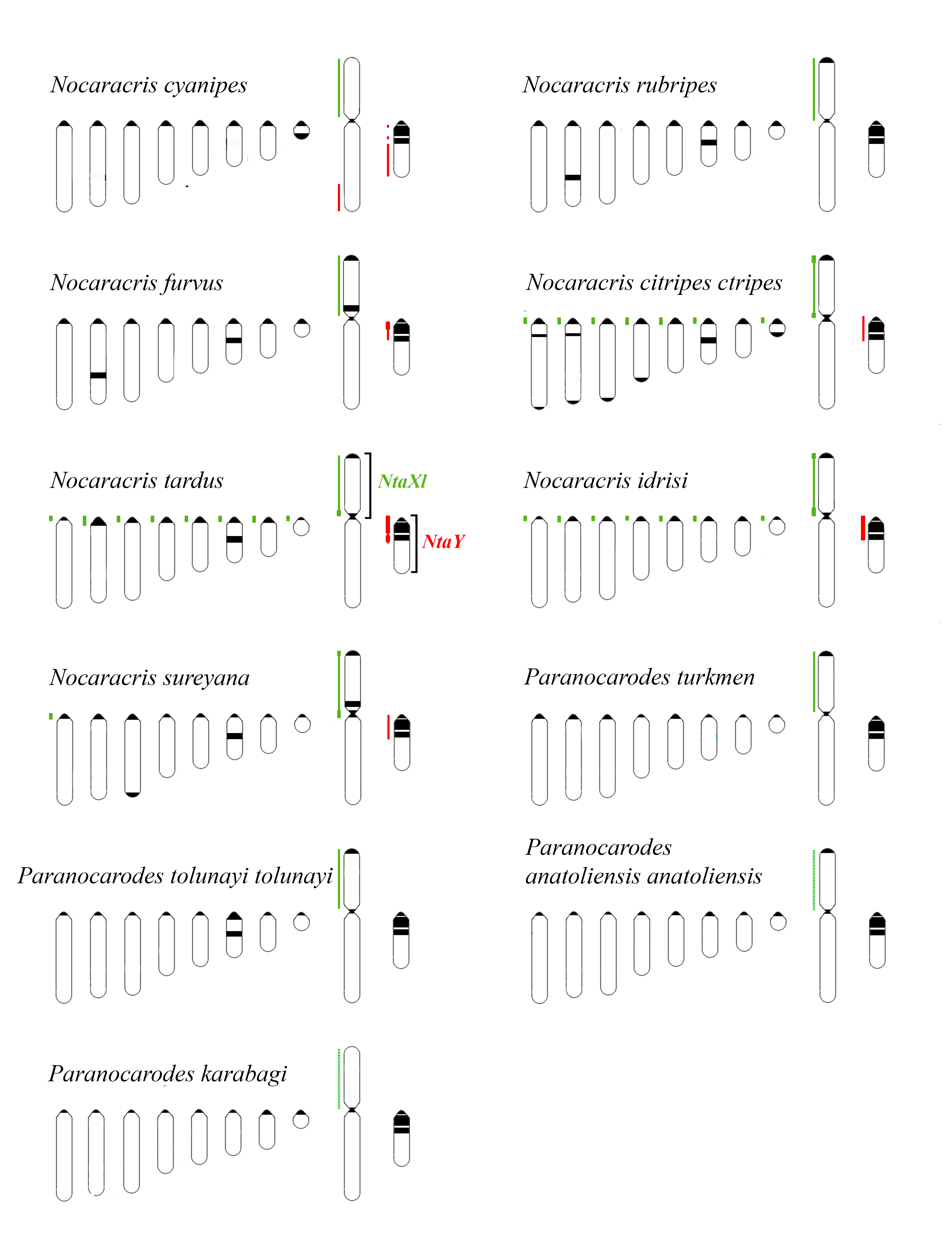

Supplement: Supplementary file 1 [file genes-08-00323-s001.zip › Fig S3.tif]

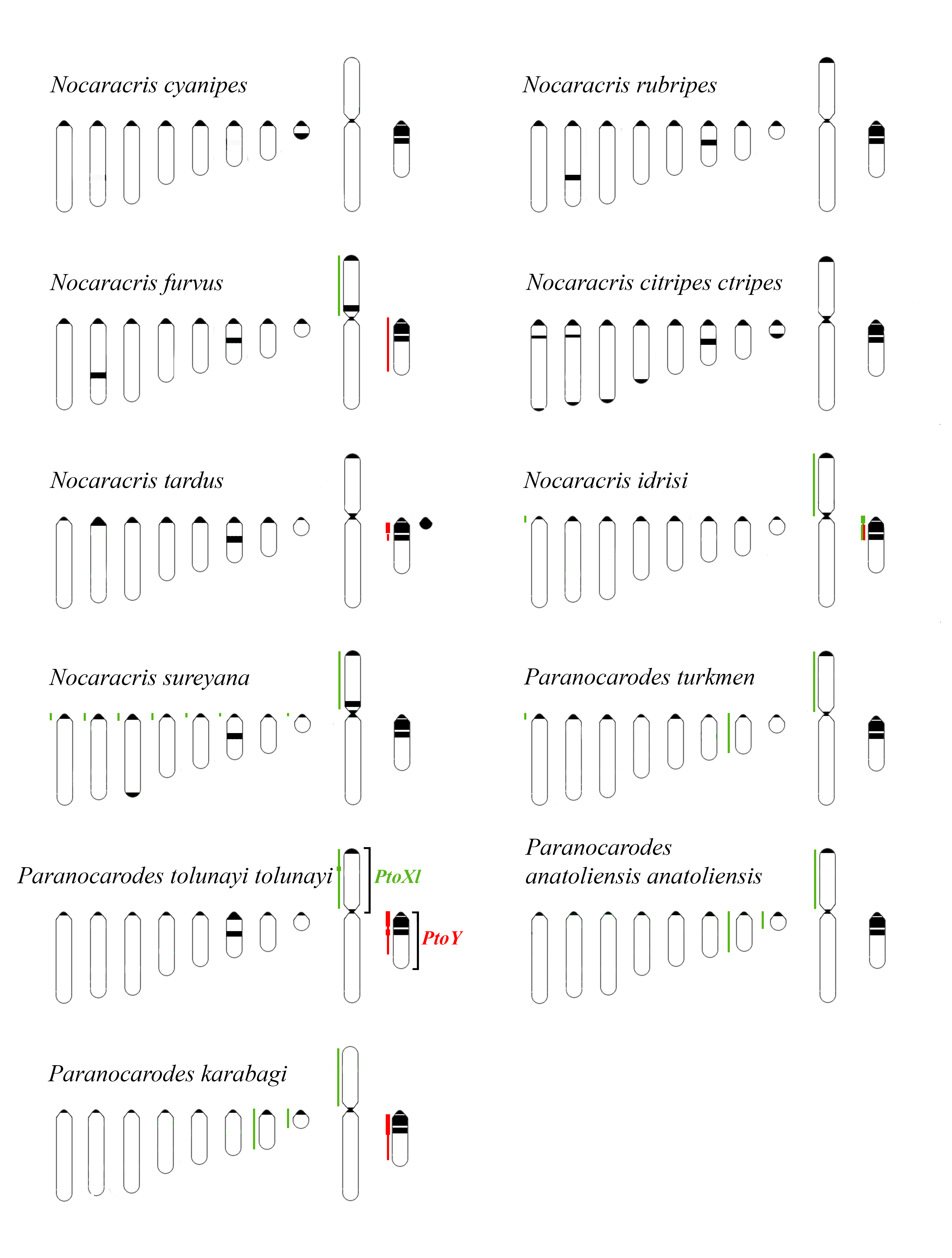

Supplement: Supplementary file 1 [file genes-08-00323-s001.zip › Fig S4.tif]

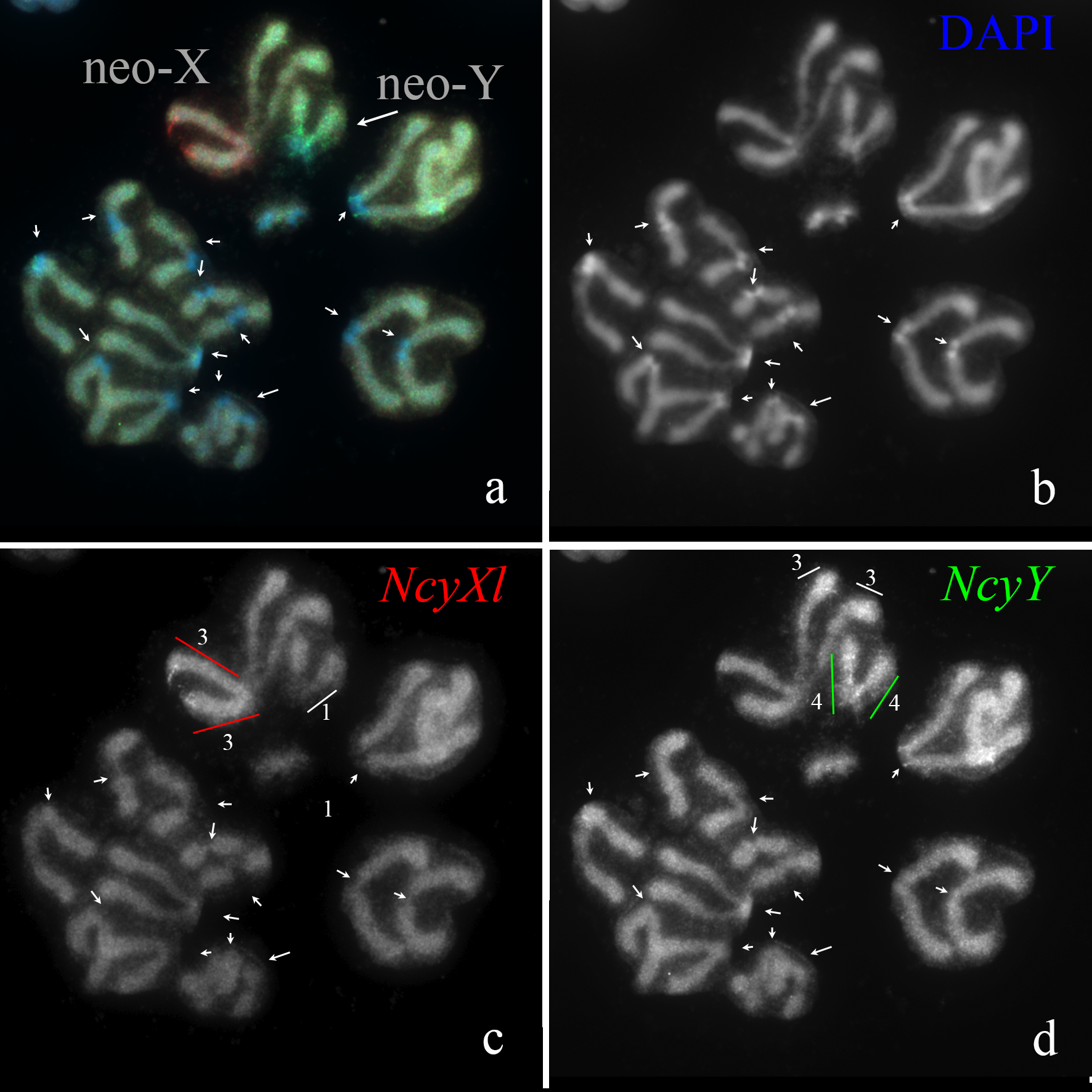

Supplement: Supplementary file 1 [file genes-08-00323-s001.zip › fig S5.tif]

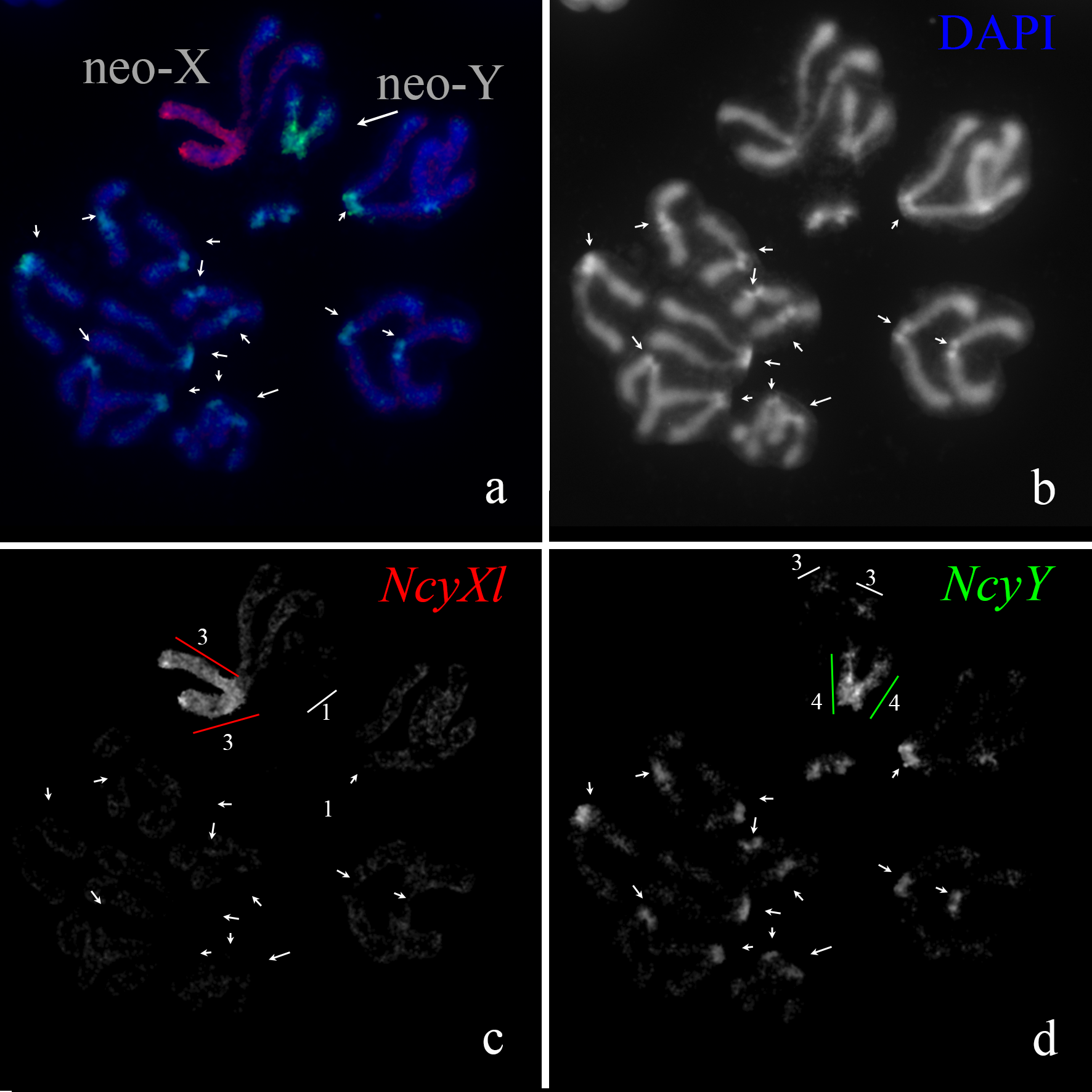

Supplement: Supplementary file 1 [file genes-08-00323-s001.zip › fig S6.tif]
